# DDX3 suppresses type I interferons and favors viral replication during Arenavirus infection

**DOI:** 10.1371/journal.ppat.1007125

**Published:** 2018-07-12

**Authors:** María Eugenia Loureiro, Andre Luiz Zorzetto-Fernandes, Sheli Radoshitzky, Xiaoli Chi, Simone Dallari, Nuha Marooki, Psylvia Lèger, Sabrina Foscaldi, Vince Harjono, Sonia Sharma, Brian M. Zid, Nora López, Juan Carlos de la Torre, Sina Bavari, Elina Zúñiga

**Affiliations:** 1 Division of Biological Sciences, University of California San Diego, La Jolla, CA, United States of America; 2 Molecular and Translational Sciences Division, United States Army Medical Research Institute of Infectious Diseases, Frederick, MD, United States of America; 3 Centro de Virología Animal, Consejo Nacional de Investigaciones Científicas y Técnicas, Buenos Aires, Argentina; 4 Department of Chemistry and Biochemistry, University of California San Diego, La Jolla, CA, United States of America; 5 La Jolla Institute for Allergy and Immunology, La Jolla, CA, United States of America; 6 The Scripps Research Institute, Department of Immunology and Microbiology, La Jolla, CA, United States of America; University of Pennsylvania School of Medicine, UNITED STATES

## Abstract

Several arenaviruses cause hemorrhagic fever (HF) diseases that are associated with high morbidity and mortality in humans. Accordingly, HF arenaviruses have been listed as top-priority emerging diseases for which countermeasures are urgently needed. Because arenavirus nucleoprotein (NP) plays critical roles in both virus multiplication and immune-evasion, we used an unbiased proteomic approach to identify NP-interacting proteins in human cells. DDX3, a DEAD-box ATP-dependent-RNA-helicase, interacted with NP in both NP-transfected and virus-infected cells. Importantly, DDX3 deficiency compromised the propagation of both Old and New World arenaviruses, including the HF arenaviruses Lassa and Junin viruses. The DDX3 role in promoting arenavirus multiplication associated with both a previously un-recognized DDX3 inhibitory role in type I interferon production in arenavirus infected cells and a positive DDX3 effect on arenavirus RNA synthesis that was dependent on its ATPase and Helicase activities. Our results uncover novel mechanisms used by arenaviruses to exploit the host machinery and subvert immunity, singling out DDX3 as a potential host target for developing new therapies against highly pathogenic arenaviruses.

## Author summary

Arenaviruses include severe pathogens causing hemorrhagic fevers and have been recently incorporated by the World Health Organization in a list of critical emerging diseases for which additional research and identification of clinical targets is urgently required. A better understanding of how viral proteins interact with host cellular factors to favor arenavirus multiplication can illuminate novel pipelines on therapeutic strategies. Here we demonstrated that the ATP-dependent RNA helicase DDX3 interacted with the arenavirus nucleoprotein, which displays fundamental functions in different steps of the viral-cycle. Our work also revealed an unexpected new biology on the role that DDX3 might play during viral infections. In sharp contrast to previous studies showing DDX3 enhancement of IFN-I induction, we demonstrated that DDX3 suppressed IFN-I production, contributing to a DDX3 pro-viral effect late after arenavirus infection. We also showed that early after infection, DDX3 pro-viral role was IFN-I independent and was mediated by facilitation of viral RNA synthesis via DDX3 ATPase and Helicase activities. Altogether, our study established DDX3 as a critical host interacting partner of the arenavirus nucleoprotein and demonstrated two previously unrecognized DDX3-dependent strategies by which these deadly viruses exploit the host cellular machinery and suppress immunity.

## Introduction

Arenaviruses include highly pathogenic hemorrhagic fever (HF) viruses endemic to West Africa and South America. Lassa virus (LASV), is an Old World (OW) arenavirus highly prevalent in West Africa where it causes about 300,000 infections and > 5,000 deaths yearly due to Lassa fever (LF), with mortality rates rising up to 50% for hospitalized patients in some outbreaks and to 90% for women in the last month of pregnancy [1,2]. Notably, increased travelling has resulted in the importation of LF cases to Europe and United States, underscoring the global risk represented by this virus [3]. Likewise, several New World (NW) arenaviruses including Junin (JUNV), Machupo (MACV), Guanarito and Sabia, as well as the more recently reported Whitewater Arroyo and Chapare viruses, cause human hemorrhagic fevers with ~30% mortality [4]. In addition, mounting evidence indicates that lymphocytic choriomeningitis virus (LCMV), a globally distributed OW arenavirus, is a neglected human pathogen that causes congenital defects and poses a special threat to immunocompromised individuals [5,6]. The live-attenuated vaccine Candid#1 has been shown to be effective against Argentine HF caused by JUNV [7], but Candid#1 is only licensed in Argentina and it does not protect against other HF arenaviral diseases. There are no other licensed arenavirus vaccines and, with the exception of the treatment with immune plasma that is restricted to JUNV infections in endemic areas [8], anti-arenaviral therapy is limited to an off-label use of the nucleoside analog ribavirin that is only partially effective [9]. Accordingly, the World Health Organization (WHO) recently included Arenaviral HF in a list of emerging diseases for which additional research and identification of clinical targets are urgently needed [10]. A better understanding of how viral proteins interact with host cellular factors to enable arenavirus propagation could aid this task.

Arenaviruses are enveloped viruses with a negative-sense RNA genome, consisting of two single-stranded segments named S (ca. 3.4 kb) and L (ca. 7.2 kb). The nucleoprotein (NP), encoded by the S segment, is the most abundant viral protein and plays critical roles in different steps of the arenavirus life cycle [4,11–14]. In addition, the arenavirus NP counteracts the host type I interferon (IFN-I) response during viral infection by preventing the activation and nuclear translocation of interferon regulatory factor 3 (IRF-3), and subsequent induction of IFN-I production [15,16]. Arenavirus-NP has also been shown to inhibit nuclear translocation and transcriptional activity of NF-κB [17]. The anti-IFN-I activity of arenavirus NP was mapped to its C-terminal region and was associated to a folding domain corresponding to a functional 3’-5’ exonuclease of the DEDDH family [18,19]. NP has also been shown to interact with IKKε [20], MDA5 and RIG-I [21] which are involved in the IRF-3 and NF-κB signaling pathways [22]. Moreover, it was recently demonstrated that JUNV NP can also subvert immune responses by associating to the dsRNA-activated protein kinase (PKR), a well-characterized antiviral protein that inhibits cap-dependent protein translation initiation via phosphorylation of eIF2α [23]. Thus, the arenavirus NP plays essential roles in viral multiplication and the virus’s ability to counteract key components of the host’s antiviral innate immune response. Targeting NP-host cell protein interactions required for NP to execute its functions could facilitate novel strategies to curtail arenavirus life cycle.

In the present study, we pursued an unbiased approach to identify novel host factors targeted by the arenavirus NP that could contribute to viral multiplication or be exploited by the virus to subvert the immune response. We found that DDX3, a DEAD (Asp-Glu-Ala-Asp)-box ATP-dependent RNA helicase, interacted with LCMV NP and was critical for supporting optimal LCMV growth, a finding that was extended to both OW and NW arenavirus infections in human cells. Strikingly, and in contrast to roles previously ascribed to DDX3 in promoting IFN-I [24,25], we observed that DDX3 contributed to IFN-I suppression upon arenavirus infection, partially explaining its pro-viral effect late in infection. In contrast, the early pro-viral effect of DDX3 was IFN-I independent and was explained by DDX3 positive effect on viral RNA synthesis. Our results uncovered previously unrecognized maneuvers evolved in highly pathogenic arenaviruses to favor their own growth by exploiting the host machinery and evading the immune system, raising DDX3 as a potential universal target for the rational design of antiviral therapies against arenaviruses infections.

## Results

### Unbiased identification of LASV and LCMV NP interacting proteins in human cells

To identify novel host proteins that could take part in protein-protein interactions with the arenavirus NP, we applied an unbiased proteomics approach. We used a human lung epithelial cell line, A549 that was transfected with plasmids expressing either LCMV or LASV NPs fused to HA tag (NP-HA). As negative controls we included cells transfected with a plasmid encoding an HA-tagged protein unrelated to the arenavirus NP but with a similar molecular weight, the ubiquitin carboxyl-terminal hydrolase 14 (HA-USP14), as well as cells infected with a newly generated recombinant tri-segmented LCMV, expressing HA-tagged GFP (3rLCMV-HA-GFP, S1A Fig). Cell lysates were immunoprecipitated with anti-HA monoclonal antibody (S1B Fig) followed by mass spectrometry to identify interacting peptides, using a criteria of at least 2 unique tryptic peptides with a degree of confidence of 99% to identify each hit. This approach revealed a number of NP-interacting host proteins for witch at least two tryptic peptides were detected with LCMV, LASV, or both, NP-HA samples but none (Table 1) or only one tryptic peptide (S1A Table) detected in negative controls. Potential NP-interacting proteins excluded due to detection in only one of the two negative control samples are shown in S1B and S1C Table. Thus, after conducting an unbiased proteomics approach in human cells, we pinpointed a selected number of host proteins as novel candidates involved in protein-protein interactions with LCMV and/or LASV NP.

**Table 1:** Host proteins interacting with the NP of LCMV, LASV, or both.

GI	Annotation	LCMV	LASV	Total	NSC
**41399285**	**Chaperonin (HSPD1)**	3	3	6	0.03892668
55956788	Nucleolin #	3	1	4	0.011823951
7705813	Ribosomal protein L26-like 1	2	1	3	0.01543909
4506633	Ribosomal protein L31 isoform 1	1	2	3	0.012745707
4506619	Ribosomal protein L24	2	1	3	0.010694274
4506623	Ribosomal protein L27	2	1	3	0.008230397
15431297	Ribosomal protein L13	1	2	3	0.007550774
4506607	Ribosomal protein L18	2	1	3	0.005953904
57863257	T-complex protein 1 isoform a	0	2	2	0.020058442
57013276	Tubulin, alpha, ubiquitous #	1	1	2	0.019521338
4502643	Chaperonin containing TCP1, subunit 6A isoform a	0	2	2	0.01500201
4506671	Ribosomal protein P2	1	1	2	0.012759599
48762932	Chaperonin containing TCP1, subunit 8 (theta)	0	2	2	0.012183282
63162572	Chaperonin containing TCP1, subunit 3 isoform a	0	2	2	0.011693309
4506703	Ribosomal protein S24 isoform c	1	1	2	0.011032736
4506701	Ribosomal protein S23	1	1	2	0.010261216
4504351	Delta globin #	2	0	2	0.010124655
5453603	Chaperonin containing TCP1, subunit 2	0	2	2	0.008933907
38455427	Chaperonin containing TCP1, subunit 4 (delta)	0	2	2	0.008867607
4506741	Ribosomal protein S7	1	1	2	0.00756368
4506667	Ribosomal protein P0	1	1	2	0.00526533
117190254	Heterogeneous nuclear ribonucleoprotein C isoform b	1	1	2	0.005008034
**156071459**	**Solute carrier family 25, member 5 (SLC25A5)**	1	1	2	0.004924006
**4758138**	**DEAD (Asp-Glu-Ala-Asp) box polypeptide 5 (DDX5)**	1	1	2	0.004779654
4506725	Ribosomal protein S4, X-linked X isoform	1	1	2	0.00423095
**4557871**	**Transferrin (TF)**	2	0	2	0.00213227
**4885661**	**Viral oncogene yes-1 homolog 1 (YES1)**	2	0	2	0.001370464
**16751921**	**Dermcidin preproprotein (DC)**	1	0	1	0.119585846
4759160	Small nuclear ribonucleoprotein polypeptide D3	1	0	1	0.017718147
**4504347**	**Alpha 1 globin (HGB)**	1	0	1	0.015721736
24307939	Chaperonin containing TCP1, subunit 5	0	1	1	0.012340921
5453607	Chaperonin containing TCP1, subunit 7 isoform a	0	1	1	0.011736379
15431288	Ribosomal protein l10a	1	0	1	0.010316443
58331185	Chaperonin containing TCP1, subunit 7 isoform b	0	1	1	0.009847254
4506743	Ribosomal protein S8	0	1	1	0.008024566
4503471	Eukaryotic translation elongation factor 1 alpha 1 #	1	0	1	0.006352181
5902102	Small nuclear ribonucleoprotein D1 polypeptide 16kda	1	0	1	0.006253464
4759156	Small nuclear ribonucleoprotein polypeptide A	1	0	1	0.005277746
**63252906**	**Tropomyosin 1 alpha chain isoform 7 (TPM)**	1	0	1	0.005240579
17105394	Ribosomal protein l23a	1	0	1	0.004703057
66912162	Histone cluster 2, h2bf #	1	0	1	0.004441802
4506685	Ribosomal protein S13	0	1	1	0.003684569
51477708	Heterogeneous nuclear ribonucleoprotein D isoform d	1	0	1	0.003543757
5174431	Ribosomal protein L10	1	0	1	0.003428397
47271443	Splicing factor, arginine/serine-rich 2 #	1	0	1	0.00336725
76496472	Ribosomal protein L3 isoform b	0	1	1	0.003143333
57863259	T-complex protein 1 isoform b	1	0	1	0.001829618
55770868	Tubulin, beta polypeptide 4, member Q #	1	0	1	0.0016905
**70906435**	**Fibrinogen, beta chain preproprotein (FG)**	1	0	1	0.001515605
15431310	Keratin 14	1	0	1	0.001185735
**87196351**	**DEAD/H (Asp-Glu-Ala-Asp/His) box polypeptide 3 (DDX3)**	1	0	1	0.000845418
**110431348**	**Deleted in colorectal carcinoma (DCC)**	1	0	1	0.000514279

Hits were considered positive when 2 unique tryptic peptides were detected in either LCMV or LASV samples and never detected in negative controls (HA-USP14 or 3rLCMV-HA-GFP). Samples belong to 4 independent experiments. Hits were first ranked in groups according to the number of times detected (6 to 1) in the 8 samples (4 LCMV + 4 LASV). Within each group, hits were ranked according to the highest NSC (Normalized spectral counts) value detected for each hit. #Hits detected in THP1 negative controls from unrelated MS studies. **Bold**: Hits selected for siRNA functional screening. GI: Gene identification (NCBI databank).

### Functional screening with NP interacting candidates singled out DDX3

**Fig 1:**
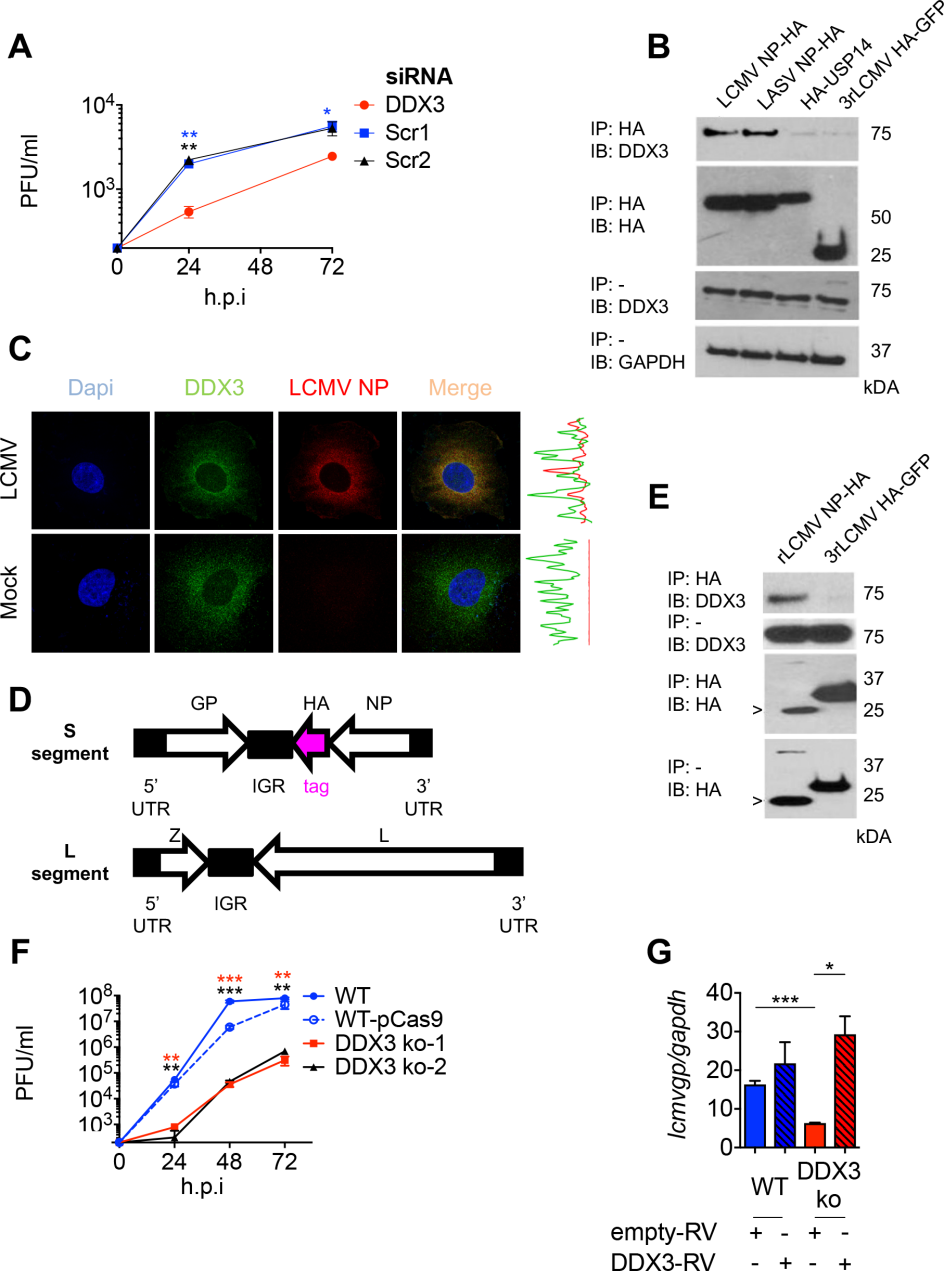
DDX3 interacted with LASV and LCMV NPs and promoted LCMV growth in human cells. **A.** A549 cells were transfected for 60h with targeting siRNAs specific for DDX3 or scrambled siRNA controls followed by infection with LCMV Cl13 (M.O.I. 0.005). Viral titers in cell culture supernatants harvested 24 and 72 h.p.i. are shown. **B.** A549 cells were transfected for 24h with plasmids encoding LCMV-NP-HA, LASV-NP-HA or HA-USP14, or infected with 3rLCMV-HA-GFP, lysed and immunoprecipitated with anti-HA agarose beads (IP HA); eluates were analyzed by Immunoblot (IB). Immunoblots with anti-DDX3 and anti-GAPDH (load control) Abs were performed in input samples. **C.** LCMV infected A549 cells (M.O.I 1) were stained for DDX3 (green), LCMV NP (red) and DAPI and processed for Confocal Microscopy. Graphs on the right represent overlapping NP and DDX3 fluorescence intensities. **D.** Schematics of the genome of rLCMV-NP-HA. White: ORFs of viral proteins. Pink: HA tag. Black: viral untranslated regions. **E.** A549 cells were infected with rLCMV-NP-HA or 3rLCMV-HA-GFP for 24h, lysed in buffer containing RNAseA 0.1 mg/ml, immunoprecipitated (IP) and analyzed by Immunoblot (IB). (>) indicates NP-HA cleaved band. **F.** Viral titers in supernatants from DDX3 ko-1, DDX3 ko-2, WT-pCas9 (control) and WT A549 cells infected with LCMV Cl13 (M.O.I. 0.5) were quantified at 24, 48 and 72 h.p.i. **G.** DDX3 ko-1 and WT A549 cells transduced with RV expressing DDX3 or empty-RV were infected with LCMV Cl13 (M.O.I. 0.5) for 24h and viral RNA levels (*lcmvgp)* were determined relative to *gapdh* by RT-qPCR and represented as relative fold expression. **(B**&**E)** Numbers on the right: MW (kDa). Data are representative of 2 (**A, E** and **G**) or 4 (**B, C** and **F**) independent experiments. * p<0.05, ** p<0.01, ***p<0.001. Stars colors represent: DDX3 vs. Scr1 (blue) or Scr2 (black) (**A**) and WT A549 vs DDX3 ko-1 (red) or vs DDX3 ko-2 (black) (**F**).

To functionally characterize the role of newly identified NP-interacting candidates in arenavirus infection, we conducted a loss-of-function assay to monitor viral growth in cells treated with small interfering RNAs (siRNAs) directed against selected NP interacting candidates (Table 1, Bold). To select these candidates, hits in Table 1 were prioritized by further filtering out proteins that were detected in THP1 negative control cells from previous unrelated mass spectrometry studies, as well as arbitrarily excluding ribosomal and ribonucleoproteins. A549 cells were incubated with targeting siRNA or scrambled siRNA (Scr1-siRNA or Scr2-siRNA) prior to LCMV infection. Approximately 90% of the cells incorporated siRNA oligonucleotides (S1C Fig) and cell viability was comparable for all siRNAs tested (S1D Fig, representative result is shown). Cells transfected with DDX3-specific, but not scrambled, siRNA showed both reduced levels of DDX3 protein expression (S1E Fig) and a reduction of LCMV titers (i.e. ~0.5 log) (Fig 1A). Overall, these results singled-out DDX3 as a host factor that could potentially play a pro-viral role in arenavirus life cycle. It is worth noting that we may have failed to identify hits for which siRNA-mediated knock-down of protein expression was not robust enough to affect LCMV multiplication.

### DDX3 associated to LCMV NP under both transfection and infection conditions

We next attempted to validate arenavirus NP interaction with DDX3. For that we transfected cells with plasmids encoding LCMV or LASV HA-tagged NPs, and as negative controls we used cells transfected with HA-USP14 or infected with 3rLCMV-HA-GFP. Cell lysates were immunoprecipitated with an anti-HA mAb, and analyzed by immunoblotting with an anti-DDX3 Ab. DDX3 levels were higher in immunoprecipates from cells transfected with LCMV or LASV NP-HA than in the negative control samples (Fig 1B, top panel). Similar levels of DDX3 and GAPDH were detected in input lysates from all samples (Fig 1B, middle and bottom panels). These results validated that DDX3 interacted with both LCMV and LASV NPs in transfected cells. Consistently with these findings, confocal microscopy analysis of LCMV-infected cells showed partial co-localization of DDX3 and NP (Fig 1C and S2 Table). To further confirm that NP and DDX3 interact in the context of arenavirus infection, we generated a recombinant LCMV expressing an HA-tagged version of NP (Fig 1D). Viral titers of rLCMV-NP-HA stocks produced in BHK-21 cells were typically ~1x10 ^7^ PFU/ml, ~1 log lower than those obtained with WT rLCMV (1x10^8^ PFU/ml), but no differences in size or shape of plaques were observed and importantly both viruses replicated to similar titers *in vivo*, revealing no gross changes in viral fitness (S1F Fig). DDX3 was immunoprecipitated from cells infected with rLCMV-NP-HA but not from control samples infected with 3rLCMV-HA-GFP (Fig 1E. top panel). In addition to full-length NP-HA (~63 kDa), and consistent with previous studies indicating arenavirus NP cleavage [26–28], we also detected a 25 kDa fragment of NP-HA in samples derived from rLCMV-NP-HA infected cells (Fig 1E, both bottom panels). DDX3 expression in input samples, as well as HA-GFP in immunoprecipitates derived from 3rLCMV-HA-GFP infected cells, were readily detectable (Fig 1E, middle panels). Together these results demonstrated that DDX3 associated with OW arenavirus NP both in transfected and infected cells.

### DDX3 promoted LCMV and LASV growth in human cells

To further investigate the role of DDX3 in OW arenavirus infection we generated two DDX3 knockout (ko) cell lines by using different non-overlapping RNA guides and CRISPR/Cas9 gene editing, and processed in parallel a control cell line transfected with a plasmid lacking RNA-guides (WT-pCas9). Immunoblot of cell lysates showed that DDX3 was undetectable (or barely detected) in both ko cell lines (S2A Fig). To investigate the impact of DDX3 gene deletion on LCMV viral growth, the DDX3 ko cell lines, WT and WT-pCas9 control cells were infected with LCMV and viral RNA synthesis and production of infectious progeny were monitored over time. Cell viability at the time of infection was similar for all cell lines (S2B Fig). Instead, production of LCMV infectious progeny was largely reduced (i.e. ~ 2 *log*) at all times examined, which correlated with reduced levels of viral RNA in DDX3 ko cells at 8 and 24 h p.i. (Figs 1F and S2C). Conversely, when DDX3 ko cells were infected with Sendai virus (SeV) we did not observe any reduction in viral RNA (S2D Fig). Importantly, reconstitution of DDX3 protein expression in DDX3 ko cells transduced with a retrovirus (RV) encoding DDX3 (S2E Fig) resulted in significantly increased viral RNA levels compared to DDX3 ko cells transduced with empty RV (Fig 1G). Overexpression of DDX3 in WT cells transduced with DDX3-RV did not however cause any significant changes in viral RNA amounts respect to WT cells transduced with empty RV (Fig 1G, blue bars). These data supported that reduced LCMV growth in DDX3 ko cells was due to lack of DDX3 expression rather than off-target effects.

**Fig 2:**
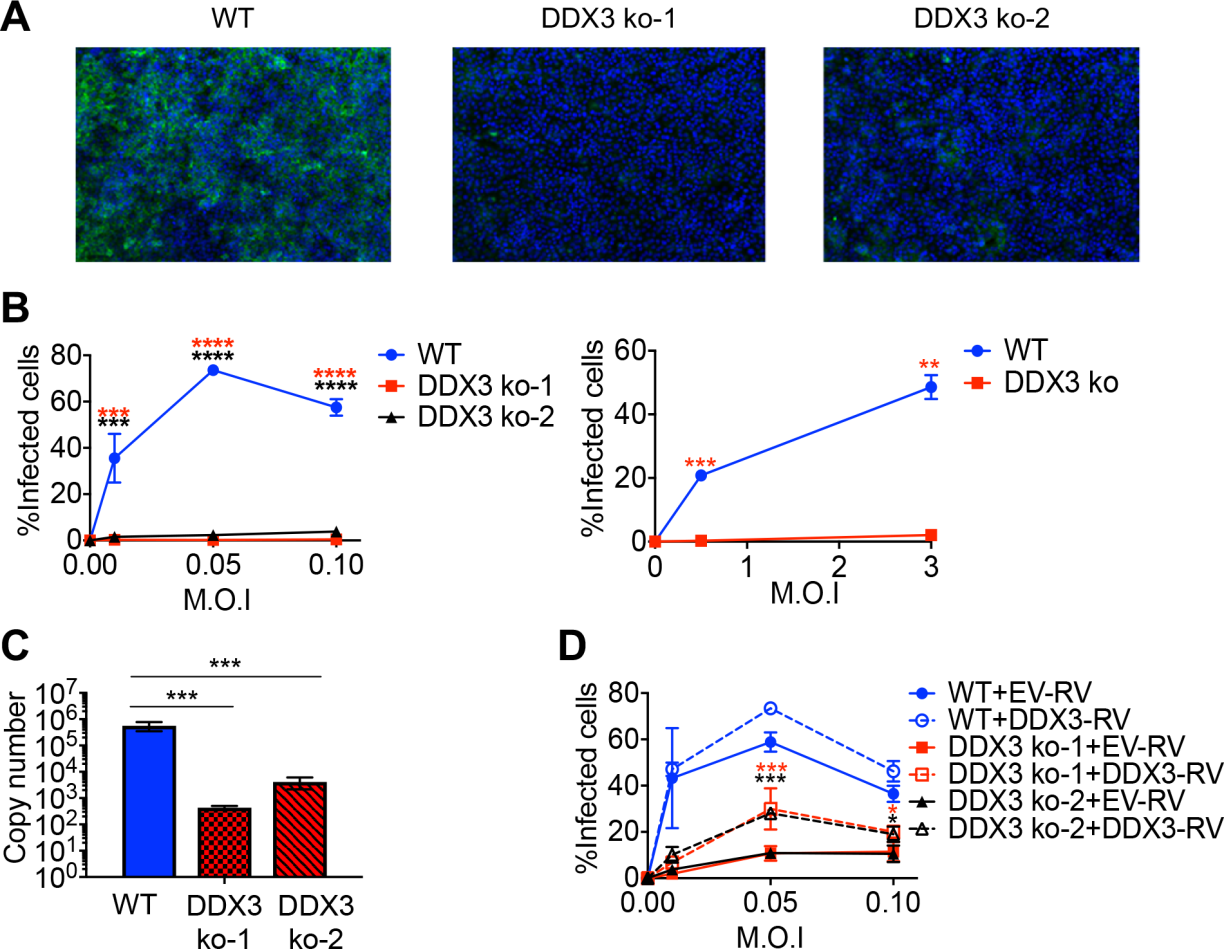
DDX3 promoted LASV growth in human cells. **A** to **C.** DDX3 ko-1, DDX3 ko-2 and WT A549 cells were infected with LASV (strain Josiah) at low M.O.I. (0.01, 0.05 and 0.1) for 48h or at high M.O.I. (0.5 and 3) for 24h. **A-B.** Cells were stained with Hoechst and anti-LASV antibodies, for confocal microscopy. **A.** Representative images are shown for infections at M.O.I. = 0.1. **B**. Percentage of infected cells were calculated by high-content quantitative image-based analysis. Left panel: GP^+^ cells at 48 h.p.i.; Right panel: NP^+^ cells at 24 h.p.i. **C.** Absolute copy numbers of viral RNA were determined in tissue culture supernatants by qRT-PCR. Representative results are shown for infections at M.O.I. = 0.01. **D.** DDX3 ko-1, DDX3 ko-2 and WT A549 cells were transduced with empty-RV (EV-RV) or RV encoding DDX3 (DDX3-RV) prior to LASV infection and then processed as in **B**. All data are representative of 2 independent experiments. * p<0.05, ** p<0.01, ***p<0.001. Star colors represent: WT-A549 vs. DDX3 ko-1 (red) or vs. DDX3 ko-2 (black) (**B**) and DDX3 ko-1+EV-RV vs DDX3 ko-1+DDX3-RV (red) or DDX3 ko-2+EV-RV vs DDX3 ko-2+DDX3-RV (black) (**D**).

We next evaluated the role of DDX3 during infection with the HF OW arenavirus LASV. For that, we performed similar infection experiments in WT versus DDX3 ko cell lines in BSL-4. Quantification of infected cells via confocal microscopy revealed a significant reduction in LASV growth in both DDX3 ko cell lines compared to WT controls at all the M.O.I. tested (Fig 2A and 2B). Consistently, we detected 100 to 1000-fold less LASV RNA in the culture supernatants of both DDX3 ko versus WT cell lines (Fig 2C). LASV infection rates were increased in both DDX3 ko cell lines when DDX3 expression was reconstituted via RV transduction, reaching statistical significance at M.O.I. 0.05 and 0.1 (Fig 2D).

These observations provided strong evidence that DDX3 promoted optimal viral growth during infection with OW arenaviruses LCMV and LASV in human cells.

### DDX3 contributed to the IFN-I suppression observed upon arenavirus infection

**Fig 3:**
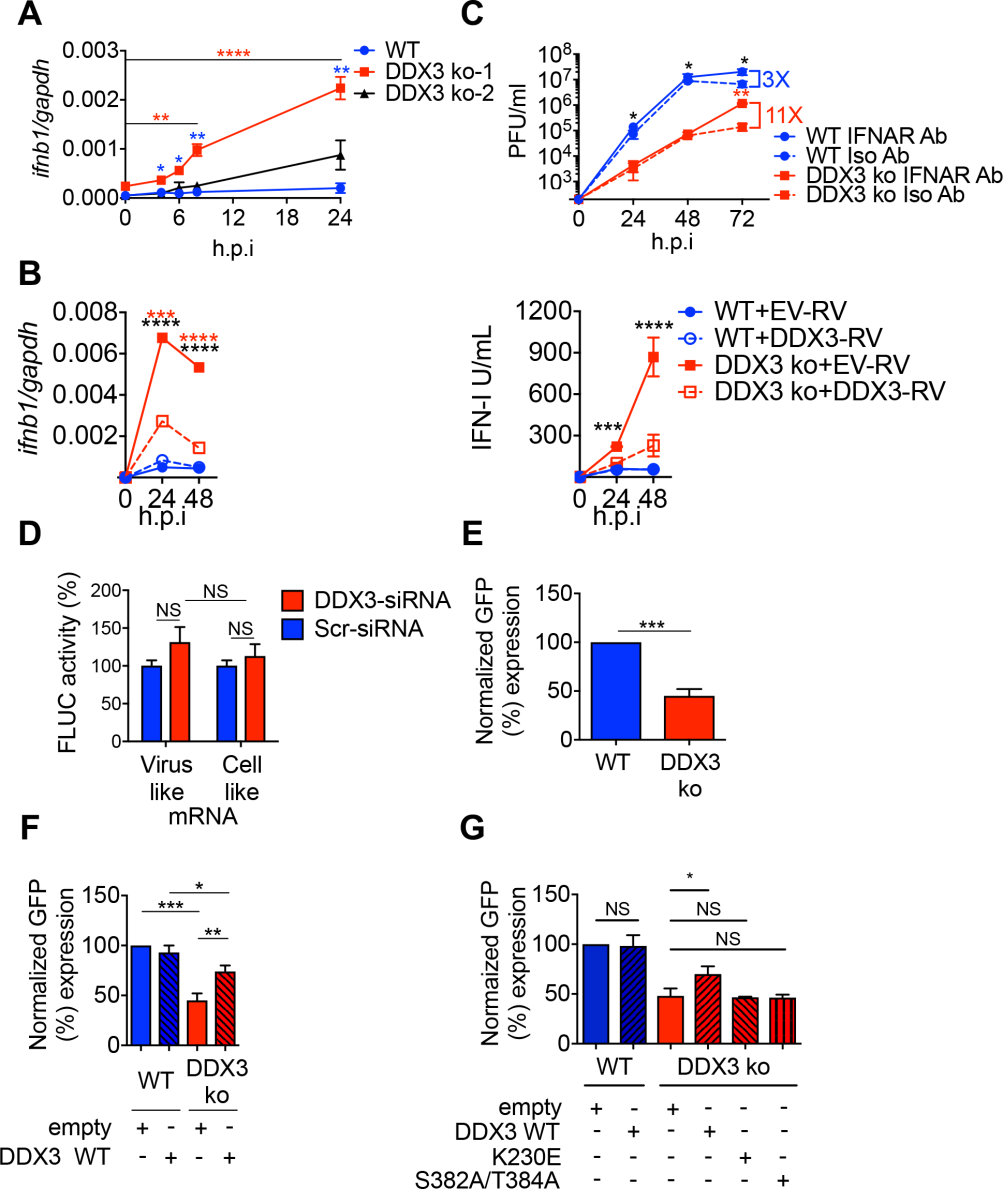
DDX3 contributed to IFN-I suppression and viral replication/transcription after arenavirus infection in human cells. A. DDX3 ko-1, DDX3 ko-2 and WT A549 cells were infected with LCMV Cl13 (M.O.I. 0.5) for the indicated times. Total RNA in cell lysates was extracted and normalized interferon transcript levels (*ifnb/gapdh)* determined by qRT-PCR and represented as relative fold expression. **B.** DDX3 ko-1 and WT A549 cells were transduced with empty-RV (EV-RV) or RV encoding DDX3 (DDX3-RV) before infection, and processed as in **A** for quantification of *ifnb/gapdh* transcripts via qRT-PCR (**B,** left panel) or determination of bioactive IFN-I levels in cell culture supernatants at indicated h.p.i. (**B,** right panel). **C.** DDX3 ko-1 and WT A549 cells were pre-incubated for 2 h and infected with LCMV Cl13 (M.O.I. 0.5) in the presence of anti-IFNAR mAb (IFNAR Ab, solid lines) or Isotype control (Iso Ab, broken lines), which were left for the remaining of the culture. Viral titers were determined at indicated h.p.i. **D**. Translation assay performed in HEK-293T cells treated with DDX3 or scrambled (Scr) siRNA. **E.** Minireplicon assay performed in DDX3 ko-1 or WT A549 cells. **F** and **G.** DDX3 ko-1 or WT cells were transfected with 0.4 μg of empty plasmid, plasmid expressing DDX3 (**F-G**) or the indicated point-mutant DDX3 (**G**) and used for minireplicon assay. 100% value was given to WT A549 transfected with empty plasmid. Data are representative of 2 (**A-C** & **E-F**) or 3 (**D & G**) independent experiments. * p<0.05, ** p<0.01, ***p<0.001. Stars represent: DDX3ko vs WT (blue) or DDX3 ko at the indicated h.p.i. versus DDX3 ko at time = 0 (red) (**A**), DDX3 ko-EV vs WT-EV (black) or vs DDX3 ko-RV-DDX3 (red) (**B**), DDX3 ko-IFNAR vs WT-IFNAR (black) or vs DDX3 ko-Isotype (red) (**C**).

DDX3 was previously shown to interact with several components of the IFN-I pathway and to enhance IFN-I production [24,25,29,30]. To investigate a putative role for DDX3 in IFN-I induction after arenavirus infection we quantified IFNβ transcript levels in WT and DDX3 ko cells after LCMV infection. Consistent with the potent capacity of arenaviruses to suppress IFN-I induction [15,16,31–33], IFNβ was undetectable in WT cells infected with LCMV (Fig 3A, blue line). Strikingly, increasing amounts of IFNβ transcript were detected in DDX3 ko cells during the first 24 hours after LCMV infection (Fig 3A, red line). While this effect was more profound at M.O.I. 0.5, it was also significant at M.O.I. 0.1 and 2.5 (S3A Fig) and it was attenuated when DDX3 levels were reconstituted in DDX3 ko cells, at both 24 and 48 h.p.i (Fig 3B, left panel). Similar results were obtained when IFN-I bioactivity was quantified in the culture supernatant at 48 h.p.i (Fig 3B, right panel) and when expression of IFN-I stimulated genes (ISGs) *MX1* and *ISG15* was determined at 24 and 48 h.p.i (S3B and S3C Fig). These findings are in sharp contrast to the previously reported role of DDX3 in promoting IFN-I induction [24,25] and revealed for the first time a suppressive role of DDX3 on IFNβ transcription, suggesting that the influence of DDX3 on IFN-I production is context dependent.

To test whether increased IFN-I levels could contribute to the diminished LCMV growth in the absence of DDX3, we incubated WT and DDX3 ko cells with anti-IFN-I receptor (IFNAR) mAb or isotype control before and throughout LCMV infection. We observed an 11-fold increase in LCMV titers at 72 (but not 24 or 48) h.p.i. in DDX3 ko cells incubated with anti-IFNAR versus isotype mAb, in contrast to a 3-fold increase when anti-IFNAR was blocked in WT cells (Fig 3C). These results suggested that while the pro-viral effect of DDX3 was partially due to IFN-I suppression at late time points after infection, the early DDX3 pro-viral activity was independent of IFN-I signaling. This was consistent with significant reduction of LCMV RNA levels following DDX3 depletion via DDX3-specific (compared to scrambled) siRNA in Vero cells, which naturally lack the IFN-I system [34] (S3D and S3E Fig).

Altogether these results pointed to an IFN-I independent, early mechanism involved in DDX3 enhancement of arenavirus propagation, and also revealed a previously unrecognized role of DDX3 as a suppressor of IFNβ transcription, partially explaining DDX3 pro-viral role late after LCMV infection.

### DDX3 promoted arenavirus replication/transcription in an ATPase and helicase dependent manner

Given that DDX3 is able to promote translation of both viral and cellular mRNAs [35,36], we next investigated whether DDX3 also played a role in arenavirus mRNA translation. For that we used a recently reported arenavirus translation assay based on capped synthetic RNAs carrying the reporter firefly Luciferase (FLUC) open reading frame [37]. Quantification of the luciferase reporter activity after transfection of a Tacaribe (TCRV) mRNA analog, or a cell-like transcript as control, in cells treated with DDX3-specific siRNA, indicated that the translation of both the viral and cellular mRNA analogs was unchanged compared to control cells transfected with scrambled siRNA (Figs 3D and S4A). These results suggested that the reduction in viral growth observed in DDX3 deficient cells was unlikely due to reduced RNA translation. Thus, we next investigated DDX3 role in arenavirus replication/transcription by using a well-established LCMV minireplicon system that assesses the activity of the intracellularly reconstituted viral ribonucleoprotein (vRNP) responsible for directing viral RNA replication and gene transcription [12]. We used a LCMV S segment-based minigenone (MG) where the *Gaussia* luciferase (Gluc) and GFP reporter genes substituted for GPC and NP genes, respectively, within the S genome RNA (MG/Gluc-GFP). Levels of GFP expression in cells transfected with MG/Gluc-GFP together with plasmids expressing the viral trans-acting factors NP and L polymerase, serve as surrogate of the vRNP activity. GFP signal showed a 60% drop in minireplicon activity in DDX3 ko cells compared to WT controls (Fig 3E), which was significantly increased when DDX3 ko cells were transfected with a DDX3-encoding, but not empty, plasmid (Fig 3F). We next attempted to further characterize the mechanism underlying DDX3 role in LCMV multiplication. To address if the enzymatic activity of DDX3 was critical for viral replication, we examined the MG activity in DDX3 ko cells that were complemented via transfection with either WT DDX3, or the mutant forms of DDX3 K230E or S382A/T384A that have been shown to lack the ability to hydrolyze ATP or unwind RNA, respectively [38,39]. WT, but none of the mutants, DDX3 increased levels of MG activity (Fig 3G), indicating a critical role for both the ATPase and Helicase activities in promoting optimal levels of LCMV RNA synthesis. Tissue culture supernatants from either WT or DDX3 ko cells with an intracellularly reconstituted active vRNP had undetectable levels (< 10 I.U./ml. of bioactive IFN-I). In addition, treatment with an anti-human IFNAR did not alter LCMV MG-directed GFP expression levels in either A549 WT or DDX3 ko cells (S4B Fig). Although we cannot exclude a potential impact of ISGs directly induced in response to IRF-3 and/or IRF-7 activation [40,41], these results ruled out a potential IFN-I-mediated inhibitory effect on the vRNP activity in our cell-based MG system. Together, these findings suggested that the pro-viral role of DDX3 on arenavirus multiplication was likely related to DDX3’s ability to promote RNA synthesis mediated by the arenavirus vRNP, and that this function of DDX3 was dependent on its ATPase and helicase activities.

### DDX3 interacted with New World arenavirus NPs and promoted JUNV growth in human cells

**Fig 4:**
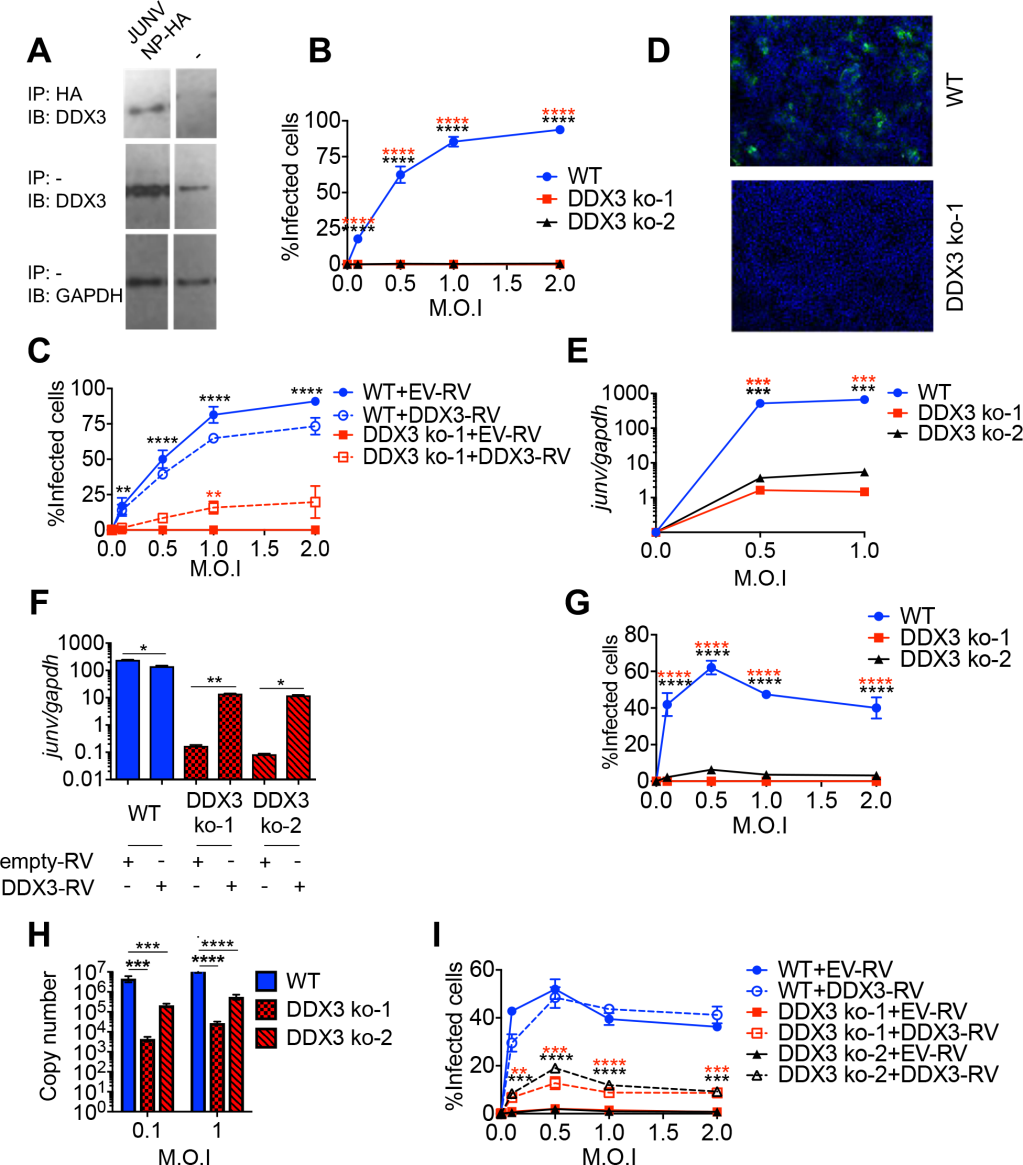
DDX3 interacted with JUNV NP and promoted JUNV growth in human cells. **A.** A549 cells were transfected with plasmid encoding JUNV NP-HA for 24h, lysed and immunoprecipitated with anti-HA agarose beads (IP HA). Eluates (upper panel) or input samples (load control, lower panel) were analyzed by Immunoblotting (IB) with anti-DDX3 or anti-GAPDH, respectively. **B-I.** DDX3 ko-1, DDX3 ko-2 and WT A549 cells were infected with JUNV Candid 1 (**B-F**) or Romero (**G-I**) strains at the indicated M.O.I. for 24h. Cells were stained with anti-GP antibodies and Hoechst, for confocal microscopy. Representative images for infected cells are shown (**D**). Numbers of infected cells were determined by high-content quantitative image-based analysis (**B**-**C** and **G and I**). Normalized viral RNA levels (*junv/gapdh*) represented as relative fold expression (**E** and **F**) and absolute copy numbers (**H**) of viral RNA in tissue culture supernatants were determined by qRT-PCR. When indicated, (**C, F and I**) DDX3 ko and WT A549 cells were transduced with empty-RV (EV-RV) or RV encoding DDX3 (DDX3-RV) before infection, and processed for confocal microscopy **(C and I)** or qRT-PCR **(F)**. Data are representative of 2 independent experiments. * p<0.05, **p<0.01,*** p<0.005, ****p<0.001. Star colors represent: DDX3 ko-EV vs WT-EV (black) or vs DDX3 ko-RV-DDX3 (red) (**C**), WT vs DDX3 ko-1 (red) or DDX3 ko-2 (black) (**E** & **G**) and DDX3 ko-1 + EV-RV vs DDX3 ko-1 + DDX3-RV (red) or DDX3 ko-2 + EV-RV vs DDX3 ko-2 + DDX3-RV (black) (**I**).

We next investigated whether DDX3-NP interaction was also conserved in NW arenaviruses. For that, cells were transfected with a plasmid expressing JUNV NP tagged with HA and cell lysates were examined by immunoprecipitation with anti-HA mAb followed by Immunoblot with anti-DDX3 Ab. DDX3 levels were significantly enriched in immunoprecipitates from cells transfected with plasmid encoding JUNV HA-tagged NP compared to cells transfected with empty plasmid (Fig 4A), indicating that DDX3 interacted with JUNV NP. We next investigated whether DDX3 also played a pro-viral role in NW arenavirus growth. For this, we first infected the two DDX3 ko cell lines (Figs 4B, 4D and S5A) with the vaccine strain of JUNV (Candid#1) [7] and monitored viral growth by confocal microscopy, using both anti-GP and anti-NP antibodies. The number of cells infected with Candid#1 was dramatically reduced in both DDX3 ko cell lines compared to WT cells at all M.O.I. tested and regardless of the antibody used for detection (Figs 4B, 4D and S5A). Importantly, DDX3 reconstitution resulted in a significant increase in infection rate, which reached statistical significance at M.O.I. 1 (Fig 4C). Likewise, we observed a ~2 log reduction in intracellular levels of Candid#1 RNA levels in DDX3 ko compared to WT cells (Fig 4E), and upon DDX3 reconstitution, levels of viral RNA in DDX3 ko cells were significantly increased (Fig 4F). However, we did not detect differences in intracellular levels of IFNβ transcripts between WT and DDX3 ko cells infected with Candid#1 (S5C Fig), not even after DDX3 protein expression restoration (S5D Fig). These results suggested that in contrast to its critical anti-IFN-I role in LCMV infection, DDX3 exerted a dispensable role in *IFNB* induction after JUNV infection. To evaluate the DDX3 role in the multiplication of a highly pathogenic strain of JUNV, both DDX3 ko cell lines were infected with JUNV Romero strain in BSL-4. Quantification of infected cells (Figs 4G and S5B) and JUNV RNA levels in cell culture supernatants (Fig 4H) indicated a dramatic decrease in both parameters in DDX3 ko versus WT control cells, regardless of the M.O.I. used. This effect was partly reverted in both DDX3 ko cell lines when DDX3 expression was reconstituted, reaching statistical significance at all M.O.I. tested (Fig 4I). These results indicated that, as with OW arenaviruses, the NP of NW arenaviruses interacted with DDX3 and that this helicase was required for optimal growth of JUNV in human cells.

## Discussion

Arenaviruses are endemic in their natural rodent hosts and often infect humans, with LASV causing thousands of lethal hemorrhagic fever cases each year [1–3]. Moreover, these viruses have been listed among the top priority emerging pathogens that are likely to cause a severe outbreak in the near future [10]. Currently there is no FDA-approved vaccine against arenavirus infections and there are limited therapeutic options that include Ribavirin, a compound with many side effects that requires administration in the first days of infection to show partial effectiveness [9]. Thus, there is an urgent need to develop new strategies to treat or prevent arenavirus infection in humans. A better understanding of arenavirus host interactions will not only inform about fundamental cellular processes exploited or subverted by these viruses, but could also help identify such intervention strategies. Given that therapeutic targeting of host (rather than viral) factors would minimize arenavirus escape mutants, we used an unbiased approach to identify arenavirus interacting candidates in human cells. Because of its abundant expression and key role in viral fitness and immune-evasion, we focused on host factors that interact with the viral protein NP. Among several newly identified NP interacting candidates, we established DDX3, an ATP-dependent RNA helicase, as an arenavirus target that is exploited to suppress host immunity and promote viral replication/transcription.

Notably, we have biochemically validated NP-DDX3 interaction in both NP-transfected and virus-infected cells, for both OW and NW arenaviruses. These results are consistent with a very recent report that examined the interactome of LCMV and JUNV NPs via mass spectrometry and documented (but did not biochemically validate or functionally characterize) DDX3 among numerous other NP interacting candidates [23]. While some of the NP-interacting proteins reported by King et al. (e.g. DDX3, DDX5, SCLC25A5, HSPD1 and TPM) were also detected in our study, other candidates were detected in only one of the two studies. These discrepancies could be related to the use of LCMV-infected cells [23] versus NP-transfected cells in our initial mass spectrometry approach, by the lower sensitivity of our method (i.e. lower spectral count values) and/or by the more stringent cut-off criteria that we used based on 99% confidence for peptide detection and absence of hits in two negative controls. Interestingly, neither we, nor King et al. [23] detected some previously documented NP-interacting host proteins including IKKε, MDA5 and RIG-I [20,21]. Most importantly, our functional studies demonstrated that DDX3 was critical for optimal arenavirus multiplication, as DDX3 inhibition via either siRNA or CRISPR/Cas9 gene editing led to a significant reduction in viral titers after infection not only with LCMV, but also with the HF arenaviruses LASV and JUNV. Thus, although targeting DDX3 should be carefully weighed in the context of its many physiological roles [42–45], our results raised the attractive possibility that treatment with DDX3 inhibitors could be a viable and broadly-effective approach to curtail viral replication and alleviate arenavirus infections in humans, either alone or in combination with ribavirin. Interestingly, DDX3 appears to represent a convergent viral target, as it has been reported to interact with multiple types of viral proteins [24,46–49]. Although the outcome of these interactions has revealed both pro-viral and antiviral effects of DDX3, the fact that DDX3 is targeted by distantly related viruses, suggests an important role for DDX3 in antiviral defense.

In other infections a pro-viral role of DDX3 has been related to distinct mechanisms [50]. While DDX3 helicase activity is crucial for Japanese encephalitis virus and norovirus replication via an unknown mechanism [51,52], upon West Nile virus infection DDX3 is known to be sequestered from stress granules and processing bodies towards viral replication sites [53]. As for human immunodeficiency virus (HIV) infection, DDX3 is important for both the nuclear export of unspliced vRNA and the translation of HIV transcripts [48,54]. DDX3 specifically represses cap-dependent translation but enhances hepatitis C virus (HCV) internal ribosome *in vitro*, leading to the proposal that DDX3 may selectively stimulate IRES-directed translation and augment HCV viral growth [55]. Here, we showed that DDX3 deletion resulted in inhibition of arenavirus multiplication that correlated with a decrease in vRNP activity as determined in a cell-based LCMV minireplicon assay. Our results also showed that both ATPase [38] and helicase [39] activities of DDX3 were involved in promoting LCMV replication. Because DDX3 is involved in dsRNA unwinding via its helicase domain [38], it is possible that NP interacts with DDX3 to recruit a helicase activity into the virus replication complex to facilitate RNA synthesis by the arenavirus polymerase. In addition, given that DDX3 is critical for stress granule (SG) formation [56], it is possible that arenavirus NP interaction with DDX3 has evolved to recruit SG (and other) proteins into the replication transcription complexes (RTC) organelles [57], thereby facilitating viral replication. However, we cannot rule out that, similarly to the situation documented for HCV [46], DDX3 role in arenavirus multiplication may not require its physical interaction with NP. Likewise, although the diminished LCMV growth in DDX3-deprived Vero cells indicated that the DDX3 pro-viral effect was at least partially independent of the IFN-I system, a possible additional role of the IFN response in inhibition of the vRNP activity remains to be elucidated. While our results from studies involving IFNAR blockade provided evidence that IFN-I levels did not contribute to the reduced LCMV minigenome activity or growth in DDX3 deprived cells early after infection, we cannot formally rule out the potential impact of a non-canonical up-regulation of antiviral ISGs (e.g. IFIT1 or ISG15), which can be directly induced in response to IRF-3 and/or IRF-7 activation [40,41].

DDX3 has been shown to promote the expression of pro-inflammatory cytokines and/or IFNβ in response to different stimuli including Poly (I:C) and SeV infection [58,59] and this activity has been proposed to mediate its antiviral effect against vesicular stomatitis virus [60,61]. Notably, DDX3 enhances IFN-I induction via interaction with IKKε or TBK1 [24,25,30] and also binds to RIG-I and MDA5 [61]. DDX3 can also act as a transcriptional regulator by interacting with IFNβ promoter [25]. Recently, DDX3 has also been reported to initiate MAVS signaling by sensing abortive RNA in HIV infected dendritic cells [60]. Strikingly, our experiments revealed a novel and unexpected role for DDX3 in suppressing IFNβ transcription upon LCMV infection. Such IFN-I suppression appeared to only partially contribute to DDX3 pro-viral activity late in infection, but this effect is expected to be amplified *in vivo* considering that studies in IFN-AR deficient animal models demonstrated critical roles played by IFN-I in promoting activation of almost all immune cells [62] and protection against arenavirus multiplication [63–65]. Although IFNAR blockade has been shown to relieve immunosuppression during chronic LCMV infection in mice, it also enhanced viral titers initially [66,67]. Furthermore, treatment with rIFNα/β promotes viral clearance after infection with an otherwise persistent LCMV variant [68].

It is possible that in the absence of DDX3 protein, arenavirus NP may not be able to access the IKKε complex, MDA5 and/or RIG-I, as has been reported in WT cells [20,21] leading to the NP failure to inhibit IFN-I production. It is also tempting to speculate that arenavirus NP exploits DDX3 to sequester DDX3-interacting proteins that participate in IFN-I induction (i.e. IKKε, TBK1, RIG-I, MDA5 and/or MAVS) [22,62], counteracting the formation of macromolecular complexes required for IFN-I synthesis. Furthermore, it has previously been shown that the N-terminus of DDX3 is necessary for IFN-I induction and that DDX3 mutants lacking this region can have IFN-I inhibitory effects [24]. Thus, it is also possible that different DDX3 regions might be involved in DDX3 facilitation of IFN-I induction [24,25,30,38,39,60,61,69,70] versus DDX3-mediated IFN-I suppression upon arenavirus infection. In addition, the possible requirement of DDX3 in the formation of RTC organelles may diminish IFN-I induction by enabling the compartmentalization of viral RNA replication and transcription; therefore limiting recognition of dsRNA intermediates by innate sensing receptors. Finally, we cannot rule out the possibility that DDX3 suppression of IFN-I response could be unrelated to its binding to arenavirus NP. In support of this possibility, DDX3 association with NP did not seem to be sufficient for suppressing IFN-I transcription, as *IFNB* induction was detected after JUNV infection and this was unaffected by DDX3 deficiency. Of note, our results were consistent with a previous report describing a distinct IFN-I response in OW versus NW arenaviruses and providing evidence that both the non-pathogenic Candid#1 and pathogenic Romero strains of JUNV are capable of activating the IFN-I pathway in human A549 cells, with higher levels of IFNβ in Candid#1 than in Romero JUNV-infected cells [71,72]. Thus, although further studies are necessary to fully understand the underlying mechanisms, it is remarkable that deletion of a single host protein (i.e. DDX3) resulted in (at least partial) counteraction of the long-evolved capacity of arenavirus to suppress IFNβ induction [15,16,33].

Our findings using an unbiased proteomic approach followed by biochemical validation in infected cells, identified DDX3 as a novel interacting partner of OW and NW arenavirus NP. Importantly, we have also uncovered two previously unrecognized DDX3-dependent strategies by which arenaviruses might counteract the host cell IFN-I response and exploit the host cellular machinery to maximize their multiplication. These findings provide the fundamental knowledge to consider DDX3 inhibitors as a potential therapeutic approach to treat infections by human pathogenic arenaviruses.

## Material and methods

### Cells

A549 (Human lung epithelial cells, ATCC CCL-185, Manassas, VA), BHK-21 (Newborn Hamster kidney fibroblast cells, ATCC CCL-10) and HEK-293T (Human epithelial kidney cells, ATCC CRL-11268) were cultured in Dulbecco’s Modified Eagle Medium (DMEM) (11965–118, Gibco, Grand Island, NY, USA) supplemented with 2 mM L-glutamine (25030081, Thermo Scientific), 50 U/mL penicillin-streptomycin (15140–163, Gibco), plus 10% heat-inactivated FBS (Lonza). BHK-21 were also supplemented with 20% Tryptose Phosphate Broth (18050039, Thermo Scientific). HEK-293T cells were supplemented with sodium pyruvate (1 mM) and non-essential amino acids (0.1 mM). HEK-Blue IFN-α/β cell line (InvivoGen) was maintained in HEK-293T media supplemented with 10μg/ml blasticidin (InvivoGen) and 200 μg/ml Zeocin (InvivoGen). Vero E6 cells (*Cercopithecus aethiops* kidney epithelial cells, ATCC CCL-81) were cultured in Minimum Eagle Medium (MEM) (11095–080, Gibco), supplemented with 2 mM L-glutamine, 50 U/mL penicillin-streptomycin and 7.5% heat-inactivated FBS. A549, BHK-21, HEK-293T and Vero E6 cell lines were originally provided by J.C. de la Torre (The Scripps Research Institute, La Jolla, CA) and HEK-Blue IFN-α/β cell line by S. Sharma (La Jolla Institute for Allergy and Immunology, La Jolla, CA)

### Viruses

LCMV Cl13 stocks were produced in BHK-21 cells and viral titers were determined by M6 well plaque assay on Vero cells. LCMV Cl13 infections were performed in BSL-2 facilities as previously described [73]. All work with highly pathogenic arenaviruses was performed at the United States Army Medical Research Institute of Infectious Diseases (USAMRIID) at Fort Detrick, Frederick, MD, USA, within maximum containment (BSL-4). JUNV Romero or Candid#1 strain and LASV Josiah strain viruses were propagated in Vero cells and viral infectivity was titrated by plaque assays as previously reported [74]. Sendai Virus infections were performed with Cantell strain.

### Generation of recombinant viruses

3rLCMV-HA-GFP was generated by modifying the previously described 3rLCMV-GFP virus [75] through the insertion of a HA-FLAG tag sequence (YPYDVPDYADYKDDDDK) in the N-terminal end of the GFP ORF (located in place of NP ORF) in one of the pol-I S vectors by multi-fragment assembly [76] using Phusion High Fidelity Polymerase (Thermofisher Scientific). For the viral rescue, BHK-21 cells (2 x 10^6^ cells per M6 well) were transfected for 5h by using 2μl of Lipofectamine 2000 (Invitrogen) per microgram of plasmid DNA. The plasmid mixture was composed of 0.8μg of pC-NP, 1μg of pC-L, 1.4μg of pol-I L and 0.8μg of each of the two pol-I S vectors. We confirmed expression of HA-GFP protein (~27kDA) in cells infected with 3rLCMV-HA-GFP by flow cytometry and Immunoblot with anti-HA Ab. Recombinant rLCMV-NP-HA was generated similarly, but using one single pol-I S vector expressing a modified NP ORF with the HA tag coding sequence on its C-terminal domain, as mentioned for 3rLCMV-HA-GFP. Primers for LCMV-HA-GFP: 1^st^ round (addition of FLAG sequence): Fragment 1 (Fr1-Fw: CGGACATCTGGTCGACCTCCAGCATCG and Fr1-Rv: GATTACAAGGATGACGACGATAAGTAAGACCCTCTGGGCCTCCCTGACTCTCCACCTCTTTCGAG) and Fragment 2 (Fr2-Fw: CTTATCGTCGTCATCCTTGTAATCCATCTTGTTGCTCAATGGTTTCTCAAGACAAATGCGCAATCAAATGC and Fr2-Rv: CGATGCTGGAGGTCGACCAGATGTCCG). 2^nd^ round (addition of HA sequence): Fragment 3 (Fr1-Fw and Fr3-Rv: TACCCTTATGATGTCCCAGATTATGCCGATTACAAGGATGACGACGATAAGGTGAGC) and Fragment 4 (Fr4-Fw: GGCATAATCTGGGACATCATAAGGGTACATCTTGTTGCTCAATGGTTTCTCAAGACAAATGCGCAATC and Fr2-Rv). Primers for rLCMV-NP-HA: Fragment 5 (Fr5-Fw: CCTACAGAAGGATGGGTCAGATTGTGACAATGTTTGAGGCTC and Fr5-Rv: TCCGGAGCCTACCCTTATGATGTCCCAGATTATGCCTAAGACCCTCTGGGCCTCCCTGACTCTCCACCTCTTTCGAGGTGG, Fragment 6 (Fr6-Fw: GGCATAATCTGGGACATCATAAGGGTAGGCTCCGGAGAGTGTCACAACATTTGGGCCTCTAAAAATTAGGTCATGTGGCAG and Fr6-Rv: GGTTGGACTTCTCTGAGGTCAGCAATGTTCAG) and Fragment 7 (Fr7-Fw: CTGAACATTGCTGACCTCAGAGAAGTCCAACC and Fr7-Rv: GAGCCTCAAACATTGTCACAATCTGACCCATCCTTCTGTAGG).

### Plasmids

pol-I S, pol-I L, pC-L, pC-NP, as well as pCAGGS plasmids encoding LASV, JUNV, MACV and TCRV NPs are described elsewhere [16]. HA-USP14: plasmid encoding ubiquitin-specific protease 14 fused to HA epitope to its N-terminal end [77]. Plasmid expressing DDX3 was constructed by inserting DDX3 cDNA in pCIneo-HA vector in EcoRI/NotI sites as previously described [78]. pSpCas9(BB)-2A-GFP construct is described in [79]. Plasmids expressing DDX3 S382A/T384A were generated by inverse PCR [80] using 5’ phosphorylated primers Fw: CTGCTTTTCCTAAGGAAATACAGATG Rv: CAGCAAACATCATAGTGTGGCGGAC) and Phusion High-Fidelity DNA Polymerase (ThermoFisher) according to manufacturer’s protocol. S382A/T384A Point mutations were confirmed by DNA sequencing.

### Mice

C57BL/6 mice were purchased from The Jackson laboratory (Bar Harbor, ME). All mice were bred and maintained in a closed breeding facility and mouse handling conformed to the requirements of the National Institutes of Health and the Institutional Animal Care and Use Guidelines of UCSD. 6–8 weeks old mice were infected i.v with 5 x 10^6^ PFU of rLCMV or rLCMV-NP-HA.

### Antibodies

Anti-HA-Tag (C29F4) Rabbit mAb #3724 (dilution 1:3500), Anti-GAPDH (14C10) Rabbit mAb #2118 (1:5000), Anti-rabbit IgG, HRP-linked Antibody (1:5000) were obtained from Cell Signaling Technologies. Anti-DDX3 Rabbit Ab (1:5000, SAB3500206) was obtained from Sigma-Aldrich. Goat anti-rabbit-AlexaFluor^488^ (#a11034) and goat-anti mouse-AlexaFluor^568^ (#a11004) conjugates were purchased from Thermo-Fisher (dilution 1:1000). Anti-IFNAR antibody (#21385–1, PBL Interferon Source) and Isotype control IgG2a antibody (#554126, BD Pharmingen Product) were both used at 5 μg/ml. Anti-LASV (anti-GP: L-52-161-6, anti-NP: L-52-2159-15) and anti-JUNV (anti-GP: GD01, anti-NP: Y-MAO3-BE06) antibodies were obtained from the US Army research Institute of Infectious Diseases (USAMRIID) archives (PMID: 20686043, PMID: 22607481).

### Immunoprecipitation and immunoblot

A549 cells (100,000 cells/ml) were plated on M12 wells. For Mass-spectrometry, four M12 plates were used for each experimental condition. Cells were transfected with 1μg of plasmid/well, encoding different arenavirus nucleoproteins fused to HA epitope in its C-terminal end (NP-HA [81] or plasmid encoding HA-USP14. Alternatively, cells were infected with 3rLCMV-HA-GFP with an m.o.i of 0.05. Media was replaced 6h later, and 24 h.p.t. cells were washed twice with PBS and lysed with 200 μl/well of Immunoprecipitation lysis buffer (Pierce IP Lysis Buffer, Thermo Scientific), supplemented with Complete EDTA-Free Protease Inhibitor Cocktail tablet (04693159001, Roche Applied Science). When indicated, samples were treated with 100 μg/mL RNAseA for 20 minutes before co-immunoprecipitation. All lysates were cleared by centrifugation at 12.000 rpm for 30 min at 4°C. After protein quantification, lysates were incubated at a ratio of 1mg lysate/50μL of resin (mouse monoclonal anti-HA antibody (clone HA-7) conjugated to agarose beads, A2095, Sigma-Aldrich), rotating overnight at 4°C. Beads were then washed 4 times with IP Lysis Buffer and 2 times with PBS. For IP:IB experiments, co-immunoprecipitated proteins were recovered with one volume of 4X Laemmli sample buffer (Bio-Rad) containing 2-ME and for Mass-Spectrometry, with 200 μl of 250μg/mL HA-peptide (I2149, Sigma Aldrich) per 50 μl of resin. Aliquots of eluates were resolved by 10% SDS-PAGE, transferred to PVDF membranes (EMD Millipore) and blocked for 1h at RT with 3% non-fat dry milk in PBS containing 0.1% Tween-20, Membranes were probed with the desired primary antibody (incubated overnight at 4°C), followed by incubation with HPR-conjugated antibody (1h at RT) and visualized using SuperSignal West Pico PLUS Chemiluminescent Substrate (34580, Thermo Scientific). Alternatively, gels were stained with Silver Quest Silver Staining Kit (LC6070, Thermo Scientific), following manufacturer’s instructions.

### Mass spectrometry

Proteins present in eluates were concentrated using Ultra-4, membrane PLGC Ultracel-PL (UFC801024, Amicon) and resuspended in TNE (50 mM Tris pH 8.0, 100 mM NaCl, 1 mM EDTA) buffer. Samples were adjusted to 0.1% RapiGest SF reagent (Waters Corp.) and boiled for 5 min, followed by addition of TCEP (Tris (2-carboxyethyl) phosphine) to 1 mM final concentration and incubation at 37°C for 30 min. Samples were carboxymethylated with 0.5 mg/ml of iodoacetamide for 30 min at 37°C, followed by neutralization with 2 mM TCEP, and digested with trypsin (trypsin:protein ratio—1:50) overnight at 37°C. RapiGest was degraded and removed by treatment with 250 mM HCl at 37°C for 1 h, followed by centrifugation at 14,000 rpm for 30 min at 4°C. The soluble fraction was applied to a C18 desalting column (Thermo Scientific, PI-87782). Desalted peptides were eluted from the C18 column into the mass spectrometer using a linear gradient (5–80%) of ACN (Acetonitrile) at a flow rate of 250 μl/min for 1h. The buffers used to create the ACN gradient were: Buffer A (98% H_2_O, 2% ACN, 0.1% formic acid, and 0.005% TFA) and Buffer B (100% ACN, 0.1% formic acid, and 0.005% TFA). Analysis of desalted-peptides was performed by ultra high-pressure liquid chromatography (UPLC) coupled with tandem mass spectroscopy (LC-MS/MS) using nano-spray ionization. Nano-spray ionization was done using a TripleTof 5600 hybrid mass spectrometer (ABSCIEX) interfaced with nano-scale reversed-phase UPLC (Waters corporation nano ACQUITY) using a 20 cm-75 micron ID glass capillary packed with 2.5-μm C18 (130) CSH beads (Waters corporation). MS/MS data were acquired in a data-dependent manner in which the MS1 data was acquired for 250 ms at m/z of 400 to 1250 Da and the MS/MS data was acquired from m/z of 50 to 2,000 Da. The Independent data acquisition (IDA) parameters were as follows; MS1-TOF acquisition time of 250 milliseconds, followed by 50 MS2 events of 48 milliseconds acquisition time for each event. The threshold to trigger MS2 event was set to 150 counts when the ion had the charge state +2, +3 and +4. The ion exclusion time was set to 4 seconds. Finally, the collected data were analyzed using Protein Pilot 4.5 (ABSCIEX) for peptide identifications. Identified proteins were considered specific when at least two or more unique tryptic peptides were detected with a degree of confidence of 99% [82], and were never present in HA-USP14 or 3rLCMV-HA-GFP negative controls. Spectral count normalization (NSC) was used to estimate the relative protein abundance as described in [83]. Hits identified in 4 independent experiments were ranked as depicted in Table 1.

### siRNA

A549 cells were transfected (in triplicate) with 100nM siRNA (siGENOME Smartpool, Dharmacon) directed to each of the 11 selected hits listed in Table 1 (Bold), using Hi-Perfect reagent (Qiagen) and media was replenished 6 hours post-transfection, according to manufacturer’s protocol. As control, cells were transfected with scrambled siRNA Pool 1 (Scr1) or Pool 2 (Scr2) (Dharmacon). siRNA transfection efficiency was evaluated using siGLO RNAi control (Dharmacon) and flow cytometry (FITC channel).

### DDX3-knockout A549 cell lines

DDX3 ko-1 and DDX3 ko-2 cell lines were generated by CRISPR/Cas9-mediated genome engineering following the protocol and algorithm described by [79] A target sequence in the first (DDX3 ko-1) or fifth (DDX3 ko-2) of human DDX3 was chosen and appropriate oligonucleotides were cloned into the BbsI site of pSpCas9(BB)-2A-GFP plasmid. (Primers: DDX3-Exon1-Fw: CACCGAGTGGAAAATGCGCTCGGGC, DDX3-Exon1-Rv: AAACGCCCGAGCGCATTTTCCACTC and DDX3-Exon5-Fw: CACCGCGGAGTGATTACGATGGCAT, DDX3-Exon5-Rv: AAACATGCCATCGTAATCACTCCGC. The plasmid was transfected for 24 hours, and the GFP-positive population was sorted by single-cell flow cytometry on a 96 well culture plate using a BD FACS Aria II Cell-Sorter. As control, A549 were transfected with empty plasmid (WT-pCas9 cells). Cells were expanded, maintained for a minimum of ten passages before their use and tested for DDX3 expression by Immunoblot with the aforementioned anti-DDX3 Ab.

### Cell viability

A549 cell viability was evaluated after knockdown with different siRNAs and after stable ko of DDX3 gene, prior to viral infection with LCMV, using Ghost-dye (Tombo Biosciences) and analysed using a BD LSRII Cytometer and FlowJo software (Treestar, Inc., Ashland, OR, USA).

### Retroviral mediated DDX3 reconstitution

DDX3 gene was cloned into pMD145, a derivative of pMD143 [84] with a P2A site instead of T2A, and retrovirus was assembled in Phoenix-AMPHO (ATCC CRL-3213) retrovirus packaging cell line after transfection with TransIT-293 transfection reagent (Mirus Bio LLC). The empty vector was also used as a negative control. After 48 hours, supernatant was collected, filtered and used for A549 transduction using 20μg/mL DEAE/Dextran. Plates were centrifuged at 1200xg for 40 minutes at room temperature and transduced cells were selected with 1.5μg/mL Puromycin.

### Colocalization analysis

A549 WT cells plated on Poly-D-lysine coated coverslips were infected or not with LCMV (MOI 1) for 24 hours, fixed with ice-cold 4% paraformaldehyde for 1h at 4°C and permeabilized with 0.3% Triton X-100. Cells were incubated overnight with primary antibodies against DDX3 and LCMV NP and for 45 min with secondary antibodies (Alexa Fluor^488^ conjugated goat anti-rabbit and Alexa Fluor^568^ conjugated goat anti-mouse, respectively). Coverslips were mounted using Prolong Gold Mountant Antifade with DAPI. Images were acquired with a Zeiss LSM 880 confocal microscope using the Fast Airyscan module and processed in ImageJ. One z-slice from each z-stack was manually selected by visually determining the maximum number of NP foci and used for analysis in ImageJ. Colocalization line scans were assessed by the methodology described in Aulas et al. (2017) [85]. Briefly, a line was drawn across a region of interest within the boundary of the cytoplasm. Intensity was measured over the line using the Plot Profile option and results were exported to Excel to generate intensity line graphs. Then intensity was plotted according to arbitrary distance for each channel. For foci co-occurrence quantification, each image was background subtracted using a rolling ball radius of 5.0 pixels, thresholded (Otsu’s Method), and binarized to identify foci location [86,87]. The total number of NP foci were counted using ImageJ’s Analyze Particles module. Afterward, masks corresponding to NP foci and DDX3 foci were multiplied to generate a mask representing overlapping foci. Overlap was defined by a minimum of 10 pixel overlap and foci were counted using ImageJ’s Analyze Particles module. For quantitative colocalization analysis, Pearson’s, Manders’, and overlap coefficients were calculated using the ImageJ JACoP plugin with automatically set thresholds [88–90].

### Quantitative image-based analysis in BSL-4 facilities

Virus-infected cells were fixed in 10% buffered formalin for 72 h and blocked in 3% bovine serum albumin-PBS for 1 h. Cells were then stained with murine mAbs against JUNV or LASV glycoprotein (GD01, L-52-161-6 antibodies, respectively,) or JUNV or LASV nucleoprotein (Y-MAO3-BE06, L52-2159-15 antibodies, respectively), at 1:1,000 dilution in blocking solution, followed by Alexa Fluor 488-conjugated goat anti-mouse IgG (1:1,000 dilution in blocking solution). All infected cells were also stained with Hoechst 33342 and HCS Cell Mask Red (ThermoFisher) for nuclei and cytoplasm detection, respectively. High-content quantitative imaging data were acquired and analyzed on an Opera confocal reader (model 3842 and 5025; quadruple excitation high sensitivity; Perkin-Elmer), at two exposures using a ×10 air objective lens as described previously [91]. Analysis of the images was accomplished within the Opera environment using standard Acapella scripts. Nuclei and cytoplasm staining were used to determine total cell number and cell borders, respectively. Mock-infected cells were used to establish a threshold for virus-specific staining. Quantification of virus positive cells was subsequently performed based on mean fluorescent intensities in the virus-specific staining channel. Infection rates were then determined by dividing the number of virus positive cells by the total number of cells measured.

### qPCR

For BSL-2 analyses total RNA was extracted using RNeasy kits (Qiagen), and reverse transcribed into cDNA using MMLV RT (Invitrogen). cDNA quantification was performed using SYBR Green PCR kits (Applied Biosystems) and a Real-Time PCR Detection System (ABI). For BSL-4 infections, viral RNA yields from the media were determined by qRT-PCR as previously described [91]. Briefly, RNA was extracted with Trizol (Thermo Fischer Scientific) and the Ambion Blood RNA Isolation Kit (Thermo Fischer Scientific). The assay was performed with RNA Ultra Sense one-step kit (Thermo Fisher Scientific) and TaqMan Probe (ABI, Thermo Fischer Scientific) following the manufacturer’s instructions. The primers used were: LCMV-GP-Fw: CATTCACCTGGACTTTGTCAGACTC, LCMV-GP-Rv: GCAACTGCTGTGTTCCCGAAA, LCMV-NP-Fw: GCATTGTCTGGCTGTAGCTTA, LCMV-NP-Rv: CAATGACGTTGTACAAGCGC; JUNV-NP-Fw: CGCCAACTCCATCAGTTCATC, JUNV-NP-Rv: CCATGAGGAGTGTTCAACGAAA; probe JUNV NP Prb: 5-6FAM- TCCCCAGATCTCCCACCTTGAAAACTG-TAMRA; LASV-GPC-Fw: GCAGTGCTGAAAGGTCTGTACAA, LASV-GPC-Rv: AGGAGGAAAGTGACCAAACCAA, probe LASV-GPC: 5-6FAM-TTTGCAACGTGTGGCCT-TAMRA; SeV-NP-Fw: TGCCCTGGAAGATGAGTTAG, SeV-NP-Rv: GCCTGTTGGTTTGTGGTAAG; huIFNb Fw: AAACTCATGAGCAGTCTGCA, huIFNb-Rv:AGGAGATCTTCAGTTTCGGAGG huGAPDH-Fw: TGATGACATCAAGAAGGTGGTGAAG and huGAPDH-Rv: TCCTTGGAGGCCATGTGGGCCAT. Serial 10-fold dilutions of the assayed (10^2^ to 10^7^ copies) virus RNA were used as standards. Relative expression levels were determined by using the comparative cycle threshold method.

### Bioactive IFN-I

Human IFN-I bioactivity in tissue culture supernatants was measured with reference to a recombinant human IFN-β standard (InvivoGen) using HEK-BlueIFN-α/β cell line (InvivoGen) and QUANTI-Blue detection reagent, following manufacturer’s instructions. In minireplicon assays, levels of bioactive IFN-I in tissue culture supernatants were determined by evaluating protection capacity against cytopathic effect (CPE) after 24h infection of Vero cells with VSV at moi = 0.1. As control, cells were treated with either 1000, 100 and 10 I.U./ml of IFN-I for 16 hours. Under these conditions, 10 I.U./ml protected against VSV induced CPE.

### Minireplicon and translation assay

LCMV minireplicon system was assayed as described elsewhere [92]. Briefly, WT and DDX3 ko A549 cells were transiently co-transfected, using Lipofectamine 2000, with 0.6 μg of pCAGGS L, 0.15 μg of pCAGGS NP and 0.5 μg of the dual-reporter (green fluorescent protein (GFP) and *Gaussia* luciferase (Gluc)) minigenome (MG) plasmid. These constructs were driven by the human polymerase-I promoter [93]. To normalize transfection efficiencies, 0.1 μg of a mammalian expression vector encoding *Cypridina noctiluca* luciferase (Cluc) under the control of the constitutively active simian virus 40 (SV40) promoter (pSV40-Cluc; New England BioLabs), were included in the transfection mix. GFP expression was determined by fluorescence microscopy using a Leica fluorescence microscope. Microscope images were pseudocolored using Adobe Photoshop CS4 (v11.0) software and by luminometry (Gluc) using a Lumicount luminometer (Packard). Cells were also subjected to flow cytometry analysis at 72 h post-transfection, and percentages of GFP-positive (GFP^+^) cells and mean fluorescence intensities (MFI) of the FL1-gated cell population were determined using FlowJo software (Tree Star). Luciferase gene activities were determined using Biolux *Gaussia* and *Cypridina* Luciferase Assay kits (New England BioLabs) using a Lumicount luminometer (Packard). Reporter gene activation (Gluc) is indicated as fold induction over cells transfected with a negative pCAGGS empty plasmid control instead of the viral NP. The translation assay was performed as indicated in [37]. Capped synthetic RNAs were obtained by *in vitro* transcription from T7 promoter-controlled constructs. The virus-like mRNA (5’wt/3’wt_2), mimicking the TCRV NP mRNA comprises a 5-nt nonviral sequence preceding the viral 5’UTR, which is fused to the reporter firefly Luciferase (FLUC) open reading frame followed by the viral 3’ UTR. The cell-like 5’βGlo/3’poly(A) transcript bears the 5’UTR from human β-globin, and a 53-nt 3’ poly(A) tail flanking the FLUC coding sequence. Briefly, HEK-293T cells were grown in 24 well plates, transfected with 50 pmol of siRNAs against DDX3 or scrambled siRNA pool 1 (Scr), and 42 hours later transfected again with 200 ng per well of the indicated capped synthetic RNA. As internal control, 75 ng/well of a Renilla Luciferase (RLUC)-expressing non-capped mRNA was added to the transfection mix. Following 6h incubation, lysis of transfected cells and quantification of FLUC and RLUC activities on a Biotek FLx800 luminometer were performed using Dual-luciferase reporter assay system (Promega), according to the manufacturer’s instructions. FLUC activity was normalized against the corresponding value of RLUC activity in each experimental condition. For each transcript, mean FLUC values (+/- standard deviation; SD) determined in depleted cells, are shown as a percentage of those in control cells, taken as 100%.

### Statistics

Statistical differences were determined by Student’s t test or by one-way or two-way analysis of variance (ANOVA) followed by Bonferroni post-hoc analysis using the GraphPad Prism 5 software (La Jolla, CA). For the Translation assay, statistical analyses were performed using the SPSS 17.0 statistical software package (SPSS, Inc., Chicago, IL, USA).

### Data availability

Mass spectrometry results were deposited in http://www.peptideatlas.org/PASS/PASS01114.

### Ethics statement

This study was carried out in strict accordance with the recommendations in the Guide for the Care and Use of Laboratory Animals of the National Institutes of Health under a protocol (S07315) approved by the Institutional Animal Care and Use Committee at the University of California, San Diego (Animal Welfare Assurance Number: D16-00020). All efforts were made to minimize suffering of animals employed in this study.

## Supporting information

S1 Fig Newly generated recombinant viruses, mass spectrometry and siRNA control assays. **A.** Schematic representation of 3rLCMV-HA-GFP genome. White: ORFs of viral proteins. Green: GFP ORF. Pink: HA-tag. Black: Viral untranslated regions. **B.** MS input samples were resolved in 10% SDS-PAGE, followed by silver-staining (left panel) or Immunoblot with anti-HA Ab (middle panel). Whole cell lysates were also probed with anti-HA Ab (right panel). Numbers on the right: MW (kDa). **C-E.** A549 cells were transfected with DDX3-specific or scrambled siRNAs (or just vehicle, Veh) for 60 h. SiRNA uptake at 6 hours post-transfection, using siGLO-siRNA (FSC: Forward scatter) (**C**) and cell viability at the time of the infection (**D**), were determined by flow cytometry; cell lysates were analyzed by Immunoblotting with anti-DDX3 Ab (IB:DDX3) or anti-GAPDH Ab (IB:GAPDH) (**E**). **F**. C57BL/6 mice were infected with 5x10 6 PFU of recombinant WT LCMV (blue) or rLCMV-NP-HA (red) (Passage 3). Serum was obtained 9 d.p.i. and viral titers determined by plaque assays.
(TIF)

S2 Fig DDX3 expression, viability and viral RNA in WT versus DDX3 ko cell lines. **A**. DDX3 ko-1, DDX3 ko-2, WT A549 and A549-pCas9 control cells were analyzed by Immunoblot with anti-DDX3 (IB:DDX3) or anti-GAPDH Ab as loading control (IB:GAPDH). **B.** Cell viability quantification at the time of the infection with LCMV Cl13. **C-D.** qRT-PCR to determine relative fold expression of viral RNA levels at the indicated h.p.i. with LCMV Cl13 **(C)** or SeV **(D)**. **E** DDX3 ko-1 and WT A549 cells were transduced with empty-RV (EV-RV) or RV encoding DDX3 (DDX3-RV), and processed as in **A**. All data are representative of 2 independent experiments. Star colors represent WT vs DDX3 ko-1 (red) or DDX3 ko-2 (Black). * p<0.05.
(TIF)

S3 Fig DDX3 suppressed IFN-I response and promoted LCMV growth in Vero Cells. **A.** DDX3 ko-1, DDX3 ko-2 and WT A549 cells were infected with LCMV Cl13 for 24 hs at the indicated M.O.I and relative fold expression of *ifnb/gapdh* transcripts were determined by qRT-PCR in cell lysates. **B-C.** DDX3 ko-1 and WT A549 cells were transduced with empty-RV (EV-RV) or RV encoding DDX3 (DDX3-RV), infected with LCMV Cl13 (M.O.I 0.5) and processed for quantification of *mx1/gapdh* and *isg15/gapdh* transcripts as in **A**. **D-E.** Vero cells were transfected with DDX3-specific or scrambled siRNAs for 60h. Cells were analyzed by Immunoblotting with anti-DDX3 (IB:DDX3) or anti-GAPDH Ab as loading control (IB:GAPDH) **(D).** Relative fold expression of viral RNA (*lcmvnp/gapdh)* was quantified via qRT-PCR after infection with LCMV Cl13 at M.O.I 0.5 for the indicated times **(E)**. All data represent 2 independent experiments. * p<0.05, ** p<0.01, ***p<0.005, ****p<0.001. Star colors represent WT A549 vs DDX3ko-1 (red) or vs DDX3ko-2 (black) (**A**); DDX3 ko-1+EV-RV vs DDX3 ko-1+DDX3-RV (black) (**B** & **C**).
(TIF)

S4 Fig DDX3 promoted early Arenavirus replication independently of IFN-I response. **A.** HEK-293T cells were transfected with DDX3-specific or scrambled siRNA for 60 hs followed by transfection with viral or cellular mRNA analogs. Cell lysates were processed for Immunoblot with anti-DDX3 (IB:DDX3) or anti-GAPDH Ab as loading control (IB:GAPDH). **B.** WT A549 (blue bars) or DDX3 ko-1 cells (red bars) were pre-incubated for 2 h with anti-IFNAR mAb (IFNAR Ab), transfected with empty plasmid or plasmid expressing DDX3 and used for minigenome assay. 100% value was given to WT A549 cells transfected with empty plasmid. Data are representative of 3 (**A**) or 2 (**B**) independent experiments.
(TIF)

S5 Fig DDX3 promoted viral growth but did not affect IFN-I production after JUNV infection. **(A-B)** DDX3 ko-1 and WT A549 cells were infected with JUNV Candid#1 (**A**) or Romero (**B**) strains for 24h at the indicated M.O.I. Cells were stained with anti-JUNV NP antibody and Hoechst and processed for confocal microscopy. Percentage of positive cells were determined by high-content quantitative image-based analysis. **C-D**. DDX3 ko-1, DDX3 ko-2 and WT A549 cells were infected with JUNV Candid#1 at M.O.I. = 0.5. In **D,** DDX3 ko-1 and WT A549 cells were transduced with empty-RV (EV-RV) or RV encoding DDX3 (DDX3-RV) before infection. *Infb* levels relative to *gapdh* were determined as relative fold expression by qRT-PCR at 48 h.p.i. Data are representative of 2 independent experiments. *p<0.05, **p<0.001. Stars colors represent: DDX3 ko vs WT (black) (**A-B**), WT vs DDX3ko-1(red) or WT vs DDX3ko-2 (black) (**C**).
(TIF)

S1 Table Proteins excluded due to detection in negative controls. List of proteins detected in at least one out of 4 LCMV or 4 LASV samples (8 samples in total) and also detected, with only 1 unique tryptic peptide in either of the two negative controls (**a**) or with ≥2 unique tryptic peptides, in HA-USP14 (**b**) or 3rLCMVGFP-HA (**c**) samples. The Normalized Spectral Counts (NSC) values were calculated for each hit in the respective negative control and the maximum value in 4 independent experiments is depicted in the sixth column (NSC). GI: Gene identity (NCBI data bank).
(DOCX)

S2 Table Pearson’s Coefficient and Overlap Coefficient for DDX3 and NP colocalization in LCMV infected cells. Pearson’s Correlation Coefficient (PCC) was used as the measure of how well red signal correlates with green signal based on linear regression. Overlap Coefficient was used as the measure of how well two fluorescence intensities overlap. Thresholded Manders Coefficient 1 and 2 (tM1 and tM2) were used as the ratio of red or green, respectively, that co-occurs with the opposite fluorescence.
(DOCX)
