# Supplementary figures and images for: DDX3 suppresses type I interferons and favors viral replication during Arenavirus infection
Source: PLoS Pathog. 2018 Jul 12;14(7):e1007125. doi: 10.1371/journal.ppat.1007125 (PMC6042795; doi:10.1371/journal.ppat.1007125)

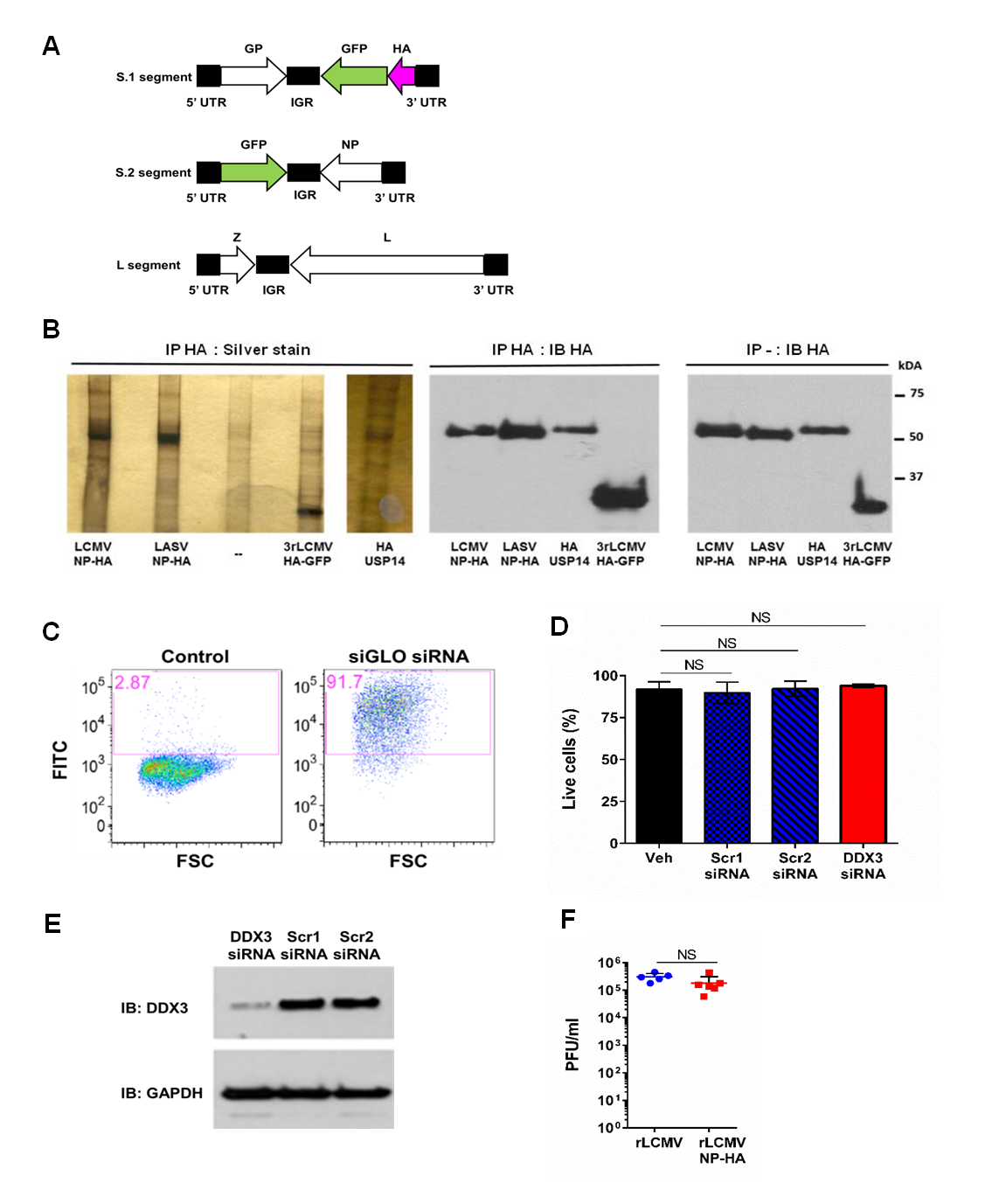

Supplement: S1 Fig — A. Schematic representation of 3rLCMV-HA-GFP genome. White: ORFs of viral proteins. Green: GFP ORF. Pink: HA-tag. Black: Viral untranslated regions. B. MS input samples were resolved in 10% SDS-PAGE, followed by silver-staining (left panel) or Immunoblot with anti-HA Ab (middle panel). Whole cell lysates were also probed with anti-HA Ab (right panel). Numbers on the right: MW (kDa). C-E. A549 cells were transfected with DDX3-specific or scrambled siRNAs (or just vehicle, Veh) for 60 h. SiRNA uptake at 6 hours post-transfection, using siGLO-siRNA (FSC: Forward scatter) (C) and cell viability at the time of the infection (D), were determined by flow cytometry; cell lysates were analyzed by Immunoblotting with anti-DDX3 Ab (IB:DDX3) or anti-GAPDH Ab (IB:GAPDH) (E). F. C57BL/6 mice were infected with 5x10 6 PFU of recombinant WT LCMV (blue) or rLCMV-NP-HA (red) (Passage 3). Serum was obtained 9 d.p.i. and viral titers determined by plaque assays. (TIF) [file ppat.1007125.s001.tif]

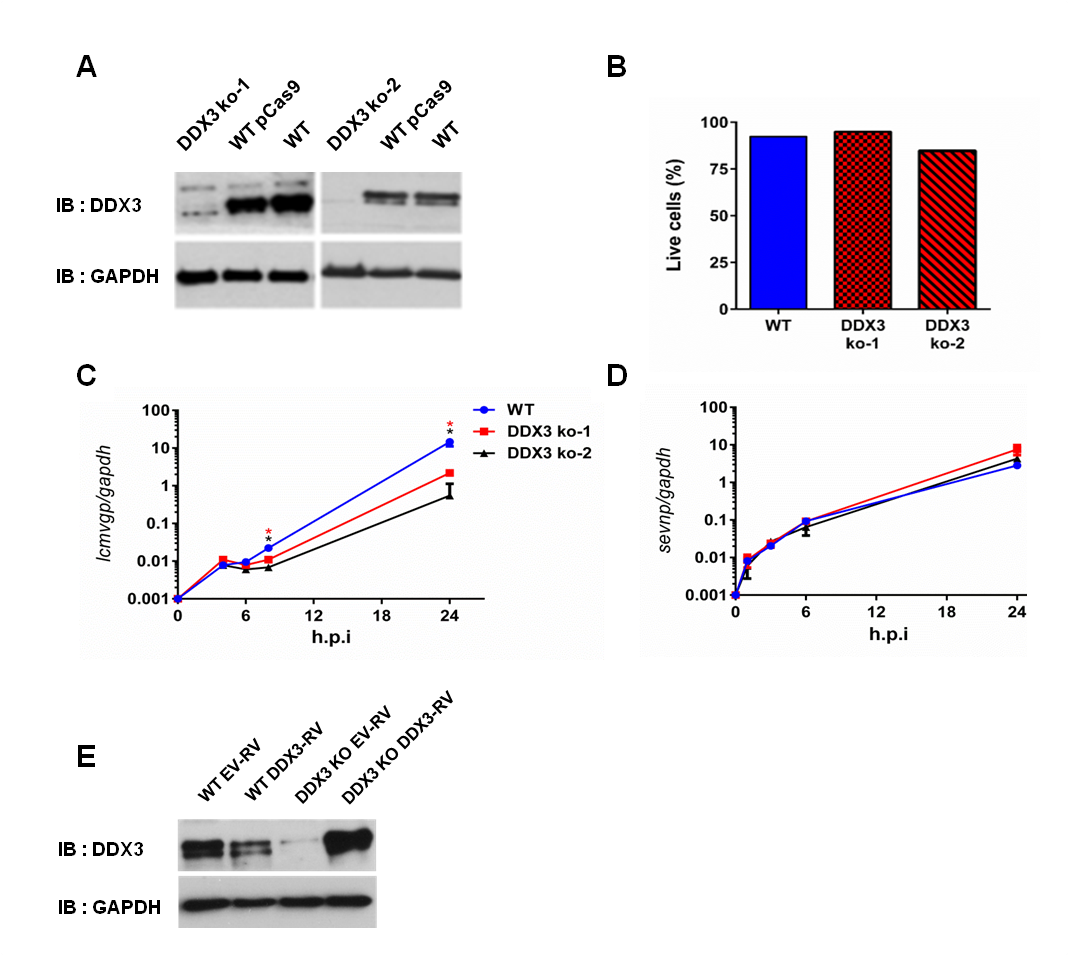

Supplement: S2 Fig — A. DDX3 ko-1, DDX3 ko-2, WT A549 and A549-pCas9 control cells were analyzed by Immunoblot with anti-DDX3 (IB:DDX3) or anti-GAPDH Ab as loading control (IB:GAPDH). B. Cell viability quantification at the time of the infection with LCMV Cl13. C-D. qRT-PCR to determine relative fold expression of viral RNA levels at the indicated h.p.i. with LCMV Cl13 (C) or SeV (D). E DDX3 ko-1 and WT A549 cells were transduced with empty-RV (EV-RV) or RV encoding DDX3 (DDX3-RV), and processed as in A. All data are representative of 2 independent experiments. Star colors represent WT vs DDX3 ko-1 (red) or DDX3 ko-2 (Black). * p<0.05. (TIF) [file ppat.1007125.s002.tif]

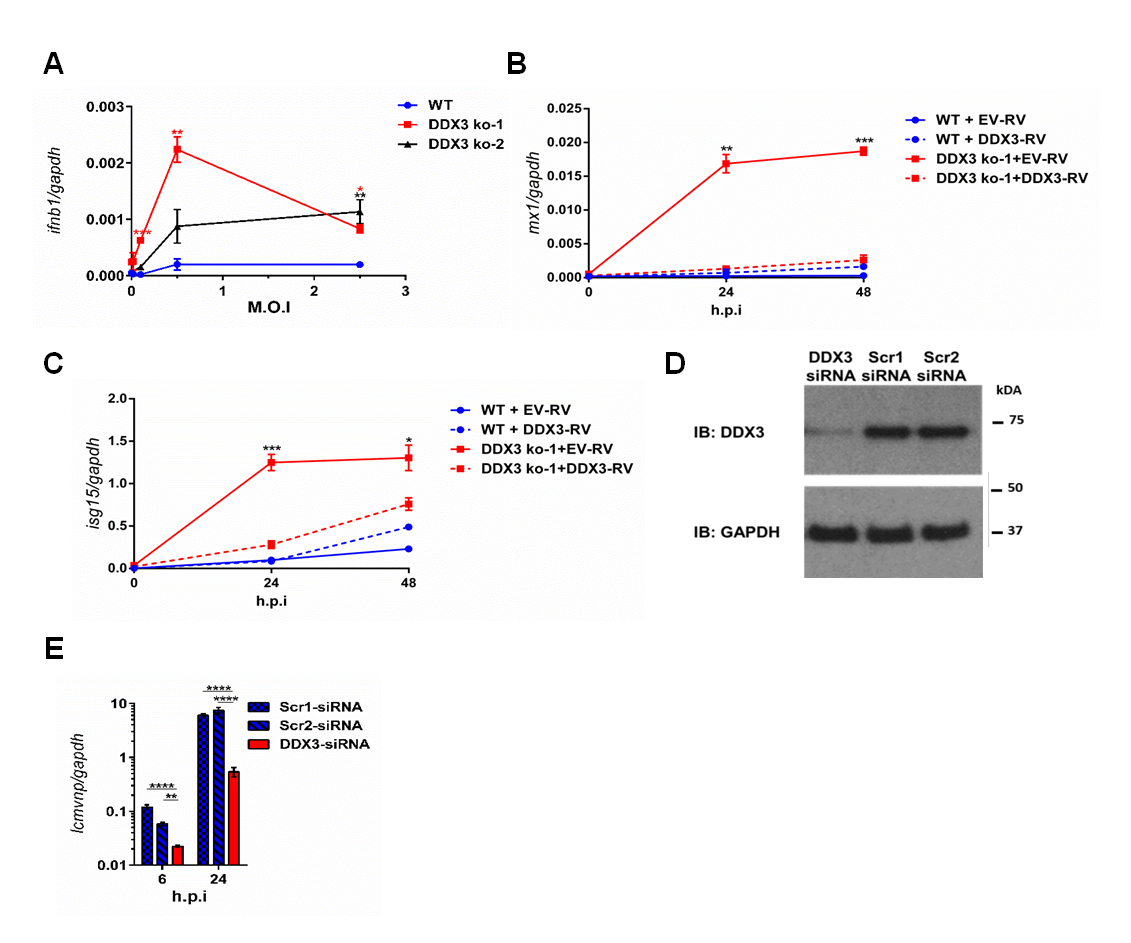

Supplement: S3 Fig — A. DDX3 ko-1, DDX3 ko-2 and WT A549 cells were infected with LCMV Cl13 for 24 hs at the indicated M.O.I and relative fold expression of ifnb/gapdh transcripts were determined by qRT-PCR in cell lysates. B-C. DDX3 ko-1 and WT A549 cells were transduced with empty-RV (EV-RV) or RV encoding DDX3 (DDX3-RV), infected with LCMV Cl13 (M.O.I 0.5) and processed for quantification of mx1/gapdh and isg15/gapdh transcripts as in A. D-E. Vero cells were transfected with DDX3-specific or scrambled siRNAs for 60h. Cells were analyzed by Immunoblotting with anti-DDX3 (IB:DDX3) or anti-GAPDH Ab as loading control (IB:GAPDH) (D). Relative fold expression of viral RNA (lcmvnp/gapdh) was quantified via qRT-PCR after infection with LCMV Cl13 at M.O.I 0.5 for the indicated times (E). All data represent 2 independent experiments. * p<0.05, ** p<0.01, ***p<0.005, ****p<0.001. Star colors represent WT A549 vs DDX3ko-1 (red) or vs DDX3ko-2 (black) (A); DDX3 ko-1+EV-RV vs DDX3 ko-1+DDX3-RV (black) (B & C). (TIF) [file ppat.1007125.s003.tif]

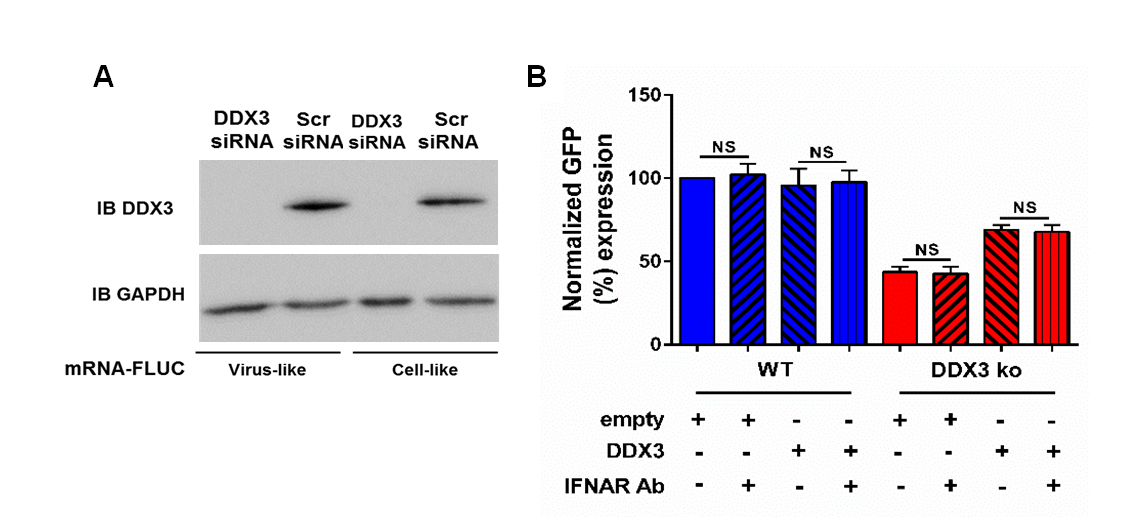

Supplement: S4 Fig — A. HEK-293T cells were transfected with DDX3-specific or scrambled siRNA for 60 hs followed by transfection with viral or cellular mRNA analogs. Cell lysates were processed for Immunoblot with anti-DDX3 (IB:DDX3) or anti-GAPDH Ab as loading control (IB:GAPDH). B. WT A549 (blue bars) or DDX3 ko-1 cells (red bars) were pre-incubated for 2 h with anti-IFNAR mAb (IFNAR Ab), transfected with empty plasmid or plasmid expressing DDX3 and used for minigenome assay. 100% value was given to WT A549 cells transfected with empty plasmid. Data are representative of 3 (A) or 2 (B) independent experiments. (TIF) [file ppat.1007125.s004.tif]

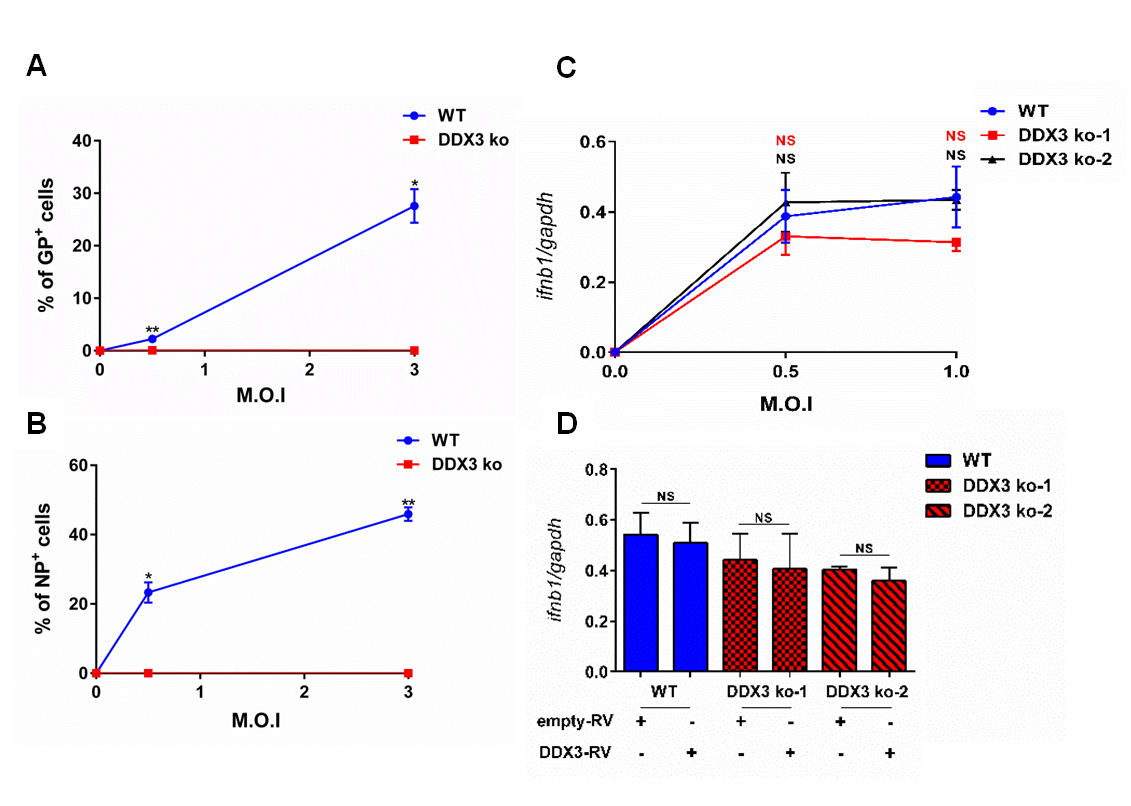

Supplement: S5 Fig — (A-B) DDX3 ko-1 and WT A549 cells were infected with JUNV Candid#1 (A) or Romero (B) strains for 24h at the indicated M.O.I. Cells were stained with anti-JUNV NP antibody and Hoechst and processed for confocal microscopy. Percentage of positive cells were determined by high-content quantitative image-based analysis. C-D. DDX3 ko-1, DDX3 ko-2 and WT A549 cells were infected with JUNV Candid#1 at M.O.I. = 0.5. In D, DDX3 ko-1 and WT A549 cells were transduced with empty-RV (EV-RV) or RV encoding DDX3 (DDX3-RV) before infection. Infb levels relative to gapdh were determined as relative fold expression by qRT-PCR at 48 h.p.i. Data are representative of 2 independent experiments. *p<0.05, **p<0.001. Stars colors represent: DDX3 ko vs WT (black) (A-B), WT vs DDX3ko-1(red) or WT vs DDX3ko-2 (black) (C). (TIF) [file ppat.1007125.s005.tif]
