# Supplementary material for: DDX3 suppresses type I interferons and favors viral replication during Arenavirus infection
Source: PLoS Pathog. 2018 Jul 12;14(7):e1007125. doi: 10.1371/journal.ppat.1007125 (PMC6042795; doi:10.1371/journal.ppat.1007125)
Supplement: S1 Table — List of proteins detected in at least one out of 4 LCMV or 4 LASV samples (8 samples in total) and also detected, with only 1 unique tryptic peptide in either of the two negative controls (a) or with ≥2 unique tryptic peptides, in HA-USP14 (b) or 3rLCMVGFP-HA (c) samples. The Normalized Spectral Counts (NSC) values were calculated for each hit in the respective negative control and the maximum value in 4 independent experiments is depicted in the sixth column (NSC). GI: Gene identity (NCBI data bank). (DOCX) [file ppat.1007125.s006.docx]

**Table S1. Proteins excluded due to detection in negative controls.**

**a. Proteins excluded due to detection of only one tryptic peptide in either 3LCMV-HAGFP or HA-USP14 negative controls**

| **GI** | | **Annotation** | | **LCMV** | | **LAS** | | **Total** | | **NSC** | |
| --- | --- | --- | --- | --- | --- | --- | --- | --- | --- | --- | --- |
| 4506901 | | Splicing factor, arginine/serine-rich 3 | | 3 | | 1 | | 4 | | 0.004537574 | |
| 4506675 | | Ribophorin I precursor | | 2 | | 1 | | 3 | | 0.001225967 | |
| 24234688 | | Heat shock 70kda protein 9 precursor | | 2 | | 0 | | 2 | | 0.014046834 | |
| 5123454 | | Heat shock 70kda protein 1A | | 2 | | 0 | | 2 | | 0.004578327 | |
| 45238849 | | Poly (A) binding protein, cytoplasmic | | 2 | | 0 | | 2 | | 0.003538014 | |
| 5031839 | | Keratin 6A | | 1 | | 0 | | 1 | | 0.013994088 | |
| 119703753 | | Keratin 6B | | 1 | | 0 | | 1 | | 0.010915491 | |
| **b. Proteins excluded due to detection of ≥ 2 tryptic peptides in HA-USP14 but never in 3LCMV-HAGFP control** | | | | | | | | | | | |
| **GI** | | **Annotation** | | **LCMV** | | **LASV** | | **Total** | | **NSC** | |
| 5901912 | | Calmodulin 1 | | 2 | | 0 | | 2 | | 0.1165681 | |
| 4504447 | | Heterogeneous nuclear ribonucleoprotein A2/B1 isoform A2 | | 0 | | 2 | | 2 | | 0.05942353 | |
| 4504445 | | Heterogeneous nuclear ribonucleoprotein A1 isoform a | | 3 | | 0 | | 3 | | 0.04553903 | |
| 25306272 | | Mitochondrial ribosomal protein L11 isoform b | | 1 | | 0 | | 1 | | 0.02508174 | |
| 4507129 | | Small nuclear ribonucleoprotein polypeptide E | | 1 | | 0 | | 1 | | 0.02262809 | |
| 4506613 | | Ribosomal protein L22 proprotein | | 1 | | 1 | | 2 | | 0.02261543 | |
| 71772415 | | Ribosomal protein s15a | | 2 | | 1 | | 3 | | 0.0222675 | |
| 100913206 | | DEAH (Asp-Glu-Ala-His) box polypeptide 9 | | 3 | | 1 | | 4 | | 0.0114744 | |
| 4826734 | | Fusion (involved in t(12;16) in malignant liposarcoma) | | 3 | | 2 | | 5 | | 0.01055404 | |
| 24234747 | | Interleukin enhancer binding factor 2 | | 1 | | 0 | | 1 | | 0.00889651 | |
| 156415990 | | Y box binding protein 2 | | 1 | | 4 | | 5 | | 0.00795268 | |
| 50592994 | | Thioredoxin | | 1 | | 0 | | 1 | | 0.00660884 | |
| 23397427 | | Synaptotagmin binding, cytoplasmic RNA interacting protein | | 3 | | 0 | | 3 | | 0.00556925 | |
| 24234756 | | Interleukin enhancer binding factor 3 isoform c | | 2 | | 0 | | 2 | | 0.00502846 | |
| 4502027 | | Albumin precursor | | 2 | | 0 | | 2 | | 0.00475332 | |
| 4501887 | | Actin, gamma 1 propeptide | | 1 | | 0 | | 1 | | 0.00370095 | |
| 14110407 | | Heterogeneous nuclear ribonucleoprotein D-like | | 1 | | 0 | | 1 | | 0.00330442 | |
| 9624998 | | Heterogeneous nuclear ribonucleoprotein H2 | | 1 | | 0 | | 1 | | 0.00309099 | |
| 55956921 | | Heterogeneous nuclear ribonucleoprotein AB isoform b | | 2 | | 0 | | 2 | | 0.00243484 | |
| 21536326 | | E1B-55kda-associated protein 5 isoform a | | 2 | | 1 | | 3 | | 0.00081066 | |
|  | |  | |  | |  | |  | |  | |
| **c. Proteins excluded due to detection of ≥ 2 tryptic peptides in 3LCMV-HAGFP but never in HA-USP14 control** | | | | | | | | | | | |
| **GI** | | **Annotation** | | **LCMV** | | **LASV** | | **Total** | | **NSC** | |
| 5031753 | | Heterogeneous nuclear ribonucleoprotein H1 | | 1 | | 1 | | 2 | | 0.02975624 | |
| 47132620 | | Keratin 2 | | 0 | | 3 | | 3 | | 0.02691947 | |
| 17978512 | | Poly-U binding splicing factor 60kda isoform a | | 1 | | 1 | | 2 | | 0.02390081 | |
| 55956899 | | Keratin 9 | | 2 | | 3 | | 5 | | 0.02208865 | |
| 4506693 | | Ribosomal protein S17 | | 0 | | 1 | | 1 | | 0.01979341 | |
| 4826760 | | Heterogeneous nuclear ribonucleoprotein F | | 1 | | 1 | | 2 | | 0.01931646 | |
| 46367787 | | Poly(A) binding protein, cytoplasmic 1 | | 1 | | 1 | | 2 | | 0.01680573 | |
| 34098946 | | Nuclease sensitive element binding protein 1 | | 2 | | 0 | | 2 | | 0.00824726 | |
| 74136883 | | Heterogeneous nuclear ribonucleoprotein U isoform a | | 2 | | 2 | | 4 | | 0.00647784 | |
| 5031755 | | Heterogeneous nuclear ribonucleoprotein R isoform 2 | | 1 | | 0 | | 1 | | 0.00422134 | |
| 5729877 | | Heat shock 70kda protein 8 isoform 1 | | 3 | | 1 | | 4 | | 0.00413639 | |
| 54112121 | | Splicing factor 3b, subunit 3 | | 1 | | 1 | | 2 | | 0.00219565 | |

List of proteins detected in at least one out of 4 LCMV or 4 LASV samples (8 samples in total) and also detected, with only 1 unique tryptic peptide, in either of both negative controls (**a**) or with ≥2 unique tryptic peptides, in HA-USP14 (**b**) or 3rLCMVGFP-HA (**c**) samples. The Normalized Spectral Counts (NSC) values were calculated for each hit in the respective negative control and the maximum value in 4 independent experiments is depicted in the sixth column (NSC). GI: Gene identity (NCBI data bank).
