# Supplementary material for: DDX3 suppresses type I interferons and favors viral replication during Arenavirus infection
Source: PLoS Pathog. 2018 Jul 12;14(7):e1007125. doi: 10.1371/journal.ppat.1007125 (PMC6042795; doi:10.1371/journal.ppat.1007125)
Supplement: S2 Table — Pearson’s Correlation Coefficient (PCC) was used as the measure of how well red signal correlates with green signal based on linear regression. Overlap Coefficient was used as the measure of how well two fluorescence intensities overlap. Thresholded Manders Coefficient 1 and 2 (tM1 and tM2) were used as the ratio of red or green, respectively, that co-occurs with the opposite fluorescence. (DOCX) [file ppat.1007125.s007.docx]

| **Sample** | **Pearson's Coefficient** | **Overlap Coefficient** | **tM1** | **tM2** |
| --- | --- | --- | --- | --- |
| 1 | 0.516 | 0.556 | 0.63 | 0.041 |
| 2 | 0.551 | 0.623 | 0.576 | 0.099 |
| 3 | 0.478 | 0.535 | 0.495 | 0.295 |
| 4 | 0.52 | 0.578 | 0.479 | 0.435 |
| 5 | 0.544 | 0.62 | 0.618 | 0.108 |
| 6 | 0.536 | 0.578 | 0.559 | 0.077 |
| 7 | 0.605 | 0.68 | 0.549 | 0.466 |
| 8 | 0.453 | 0.523 | 0.598 | 0.042 |
| 9 | 0.377 | 0.525 | 0.471 | 0.041 |
| 10 | 0.559 | 0.599 | 0.664 | 0.103 |
| 11 | 0.536 | 0.65 | 0.549 | 0.283 |
| 12 | 0.516 | 0.638 | 0.531 | 0.043 |
| 13 | 0.276 | 0.381 | 0.556 | 0.045 |
| 14 | 0.663 | 0.768 | 0.433 | 0.44 |
| Average | 0.509285714 | 0.589571429 | 0.550571429 | 0.179857143 |
| Standard Error | 0.025235904 | 0.023975655 | 0.017458181 | 0.044550115 |

**Table S2. Pearson’s Coefficient and Overlap Coefficient for DDX3 and NP colocalization in LCMV infected cells.**

Pearson’s Coefficient (PCC) was used as the measure of how well red signal correlates with green signal based on linear regression. Overlap Coefficient was used as the measure of how well two fluorescence intensities overla. Thresholded Manders Coeficient 1 and 2 (tM1 and tM2) were used as the percentage of red or green, respectively, co-occurs with the opposite fluorescence.
